# A comprehensive review of graphene-based biosensors: Fabrication, applications, characterization and future perspectives—A review

**DOI:** 10.1063/5.0266596

**Published:** 2025-09-17

**Authors:** Yao-Tung Wang, Arvind Mukundan, Riya Karmakar, Tsung-Hsien Chen, Hardik Dhiman, Fan-Min Lin, Hsiang-Chen Wang

**Affiliations:** 1 Division of Pulmonary Medicine, Department of Internal Medicine, Chung Shan Medical University Hospital, No. 110, Section 1, Jianguo North Road, Taichung City 40201, Taiwan; 2 School of Medicine, Chung Shan Medical University, No. 110, Section 1, Jianguo North Road, Taichung City 40201, Taiwan; 3 Department of Mechanical Engineering, National Chung Cheng University, 168, University Rd.., Min Hsiung, Chia Yi 62102, Taiwan; 4 Department of Biomedical Imaging, Chennai Institute of Technology, Sarathy Nagar, Kundrathur, Malayambakkam, Chennai, Tamil Nadu 600069, India; 5 Department of Internal Medicine, Ditmanson Medical Foundation Chia-Yi Christian Hospital, Chiayi 60002, Taiwan; 6 Department of Computer Science Engineering, Chandigarh University, NH-05, Ludhiana, Highway, Chandigarh, Punjab 140413, India; 7 Division of Chest Medicine, Kaohsiung Armed Forces General Hospital, 2, Zhongzheng 1st. Rd., Kaohsiung City 80284, Taiwan; 8 Department of Medical Research, Dalin Tzu Chi Hospital, Buddhist Tzu Chi Medical Foundation, No. 2, Minsheng Road, Dalin, Chiayi 62247 Taiwan; 9 Department of Technology Development, Hitspectra Intelligent Technology Co., Ltd., Kaohsiung 80661, Taiwan

## Abstract

Graphene is a 2D material that has emerged as a versatile and advanced material for biosensing technology due to its large surface area, high conductivity, and biocompatibility. These properties make it well-suited for label-free detection of biomarkers with high sensitivity and accuracy, which is crucial for early diagnosis of various diseases, environmental monitoring, and food safety. This review highlights recent progress in graphene-based biosensor technologies, emphasizing key fabrication methods such as mechanical exfoliation, liquid phase exfoliation, chemical vapor deposition, electrochemical exfoliation, and microwave-assisted exfoliation, which are highly effective and suitable for generating graphene at an industry level. Furthermore, the study highlights characterization techniques such as Raman spectroscopy, optical spectroscopy, scanning electron microscope, transmission electron microscopy, and atomic force microscopy, which ensure quality and functionality of the graphene in biosensing applications. While hurdles like enhancing conductivity and achieving large-scale production remain, graphene-based biosensors offer a transformative approach to delivering precise and consistent results across various industries, paving the way for innovative solutions in diagnostics and monitoring systems.

## I. INTRODUCTION

The development of biosensing technologies is accelerating in various fields such as food safety, environmental monitoring, the pharmaceutical industry, and healthcare, especially in the early detection of multiple diseases. The increase in chronic, neurogenerative, and infectious diseases requires effective diagnostic tools, an area that must come up with affordable, quick, and sensitive solutions for early disease prevention. Conventional diagnostic methods are very expensive, invasive, with low sensitivity, thus not well-suited for early detection. Graphene-based biosensors are much more advanced and offer efficient, portable, and accessible diagnosis.^1^ Graphene is a 2D material that consists of only one layer of carbon atoms with transmission electron microscopy (TEM) order surface area, electrical conductivity, and excellent biocompatibility. Thus, graphene is considered one of the most suitable materials for biosensing applications. These properties show that graphene reacts easily with different biomolecules, enhancing the performance of biosensors such as sensitivity and response times.^2^ With its structural versatility, graphene is used in field-effect transistor-based biosensors, surface plasmon resonance sensors, and electrochemical sensors, varied designs that leverage the strengths of graphene for specific diagnostic needs.^3^ Graphene biosensors have been involved in healthcare diagnostics; recent developments have now shown that graphene biosensors can detect biomarkers of diseases such as iron deficiency anemia, Parkinson's disease, and viral infections. Oshin *et al.* fabricated a noninvasive FET biosensor based on graphene functionalized with anti-ferritin antibodies for the detection of ferritin levels in saliva, enabling the early diagnosis of iron deficiency, a feature of particularly great value in pediatric applications.^4^ Kujawska *et al.* studied how graphene biosensors can effectively trace the biomarkers of the neurodegenerative disorder Parkinson's disease–dopamine levels–enabling early diagnosis and management of the disease.^5^

In addition to health care, graphene-based biosensors are finding huge potential in environmental monitoring and ensuring food safety. The high sensitivity and stability of graphene biosensors make them ideal for detecting environmental pollutants such as heavy metals and pesticides for safer ecosystems and food sources. Graphene-based biosensors can detect the presence of certain pathogens in much less time, thereby helping in quality and safety standards from farm to table.^2^ There are several types of graphene, each with unique characteristics and uses in biosensing. These consist of graphene quantum dots (GQDs), reduced graphene oxide (rGO), graphene oxide (GO), and virgin graphene. While GO offers a wealth of oxygen-containing functional groups that improve its dispersibility and chemical reactivity, pristine graphene delivers remarkable mechanical strength and electrical conductivity. rGO is appropriate for electrochemical biosensors because it maintains partial conductivity while improving surface functionality. Because of their edge effects and quantum confinement, GQDs have exceptional photoluminescence qualities that make them useful for fluorescence-based detection. Graphene is frequently functionalized with biomolecules (such as antibodies and DNA), polymers (such as PEG and chitosan), or nanoparticles (such as AuNPs and AgNPs) to increase its biocompatibility, selectivity, and stability. These functionalization techniques expand the biological applicability of graphene-based systems by improving sensor performance and enabling particular interactions with target analytes.

In addition, excellent biocompatibility and chemical stability make graphene an even better candidate for detecting a wide range of analytes in complex environments with no performance sacrifice.^6^ Recent advances in graphene biosensors have focused on vast improvement regarding sensitivity and limit of detection, and integration with portable devices toward point-of-care applications. Karki *et al.* proposed a highly sensitive graphene-based surface plasmon resonance (SPR) biosensor for hemoglobin detection in diagnosing anemia and polycythemia by analyzing blood samples. The research depicts the potential of graphene for label-free clinical detection methods.^7^ Material functionalization has also enabled the detection of a wider range of biomarkers, Rodrigues de Almeida *et al.*, during the designing of a graphene-based FET biosensor for the detection of anti-HIV proteins, obtained very impressive selectivity and sensitivity, enabling its applicability in HIV prevention and monitoring.^8^ Another frontier is the integration of graphene-based biosensors into wearable and smartphone technologies, including recent advances in biosensors that can be used with smartphones, capable of real-time tests accessible to the patient.^5^ Similarly, graphene-based nano biosensors, whereby salivary biomarkers might be detected to enable diagnostics of health conditions in noninvasive and real-time scenarios. Such sensors are suitable for early diagnosis undertaken from saliva and are an easy option compared to traditional invasive methods.^9^

The distinctive structure of graphene—a monolayer of sp^2^-hybridized carbon atoms organized in a honeycomb lattice—results in remarkable electrical, mechanical, and optical characteristics that are particularly beneficial for biosensing applications. The material provides an extensive specific surface area for biomolecule immobilization, rapid electron mobility for signal enhancement, and significant chemical and thermal durability, thereby facilitating reliable sensing in intricate situations. These attributes have facilitated the advancement of a diverse array of biosensors, chiefly classified into electrical, electrochemical, and optical categories. Each biosensor variant utilizes distinct properties of graphene to enhance performance regarding sensitivity, specificity, real-time functionality, and mobility, as shown in Table I. Electrical biosensors, particularly those employing field-effect transistor (FET) topologies, leverage the semiconducting characteristics of graphene to identify target molecules by monitoring variations in conductance or current flow. In a conventional graphene field-effect transistor (GFET), graphene serves as the channel material situated between the source and drain electrodes. When biomolecules, including proteins, DNA, or antigens, attach to receptors on the graphene surface, the ensuing charge redistribution modifies the local electric field, hence influencing the conductivity of the channel. Graphene's exceptional carrier mobility (∼200 000 cm^2^/V s) and its ultra-thin, atomically exposed surface render it very sensitive to minimal electrostatic variations, facilitating femtomolar-level detection of analytes without labeling requirements. Furthermore, its biocompatibility facilitates functionalization with aptamers, antibodies, and peptides without jeopardizing sensor stability. GFETs have been effective in identifying illness biomarkers (e.g., prostate-specific antigen, miRNA), viruses, and single-nucleotide polymorphisms. Their absence of labeling, minimal detection threshold, and capacity for real-time reaction render them exceptionally appealing for point-of-care diagnostics and tailored treatment. Electrochemical biosensors transmute a biological recognition event into a quantifiable electrical signal—usually current, voltage, or impedance. The electrode surface, when enhanced with graphene or its derivatives (such as graphene oxide or reduced graphene oxide), experiences an augmentation in active surface area and a facilitation of electron transport kinetics. These improvements stem from graphene's *π*-conjugated architecture and exceptional conductivity, which enable fast redox reactions at the sensor interface. Graphene can be functionalized with enzymes, metallic nanoparticles (e.g., Au and Pt), or polymers to enhance selectivity and stability. In enzymatic glucose sensors, graphene serves as a stable framework for the immobilization of glucose oxidase, simultaneously increasing the signal-to-noise ratio through increased electron transport. Electrochemical graphene biosensors have been extensively utilized for the detection of tiny biomolecules (glucose, uric acid, and dopamine), pathogens, heavy metals, and cancer biomarkers. Their affordability, straightforward equipment, and excellent reproducibility render them appropriate for portable diagnostic kits and wearable health monitors. Optical biosensors identify variations in optical signals—such as absorbance, fluorescence, surface plasmon resonance (SPR), or Raman scattering—upon interaction with certain analytes. Graphene's capacity to suppress adjacent fluorophores through Förster Resonance Energy Transfer (FRET) improves contrast and signal selectivity in nucleic acid detection. Graphene enhances the electromagnetic field at the surface when combined with metallic nanostructures, hence amplifying Raman signals from target molecules. Graphene layers applied to plasmonic metals enhance resonance sensitivity and facilitate the stable immobilization of biomolecules. Optical graphene biosensors have exceptional selectivity and are ideally suited for multiplex tests and imaging applications. They are progressively utilized for the detection of poisons, environmental contaminants, infectious agents, and allergens, frequently at attomolar concentrations. In addition to the primary categories, graphene has demonstrated potential in mass-sensitive sensors (e.g., quartz crystal microbalance and microcantilever sensors) by enhancing the resonance shift upon biomolecular adsorption owing to its substantial mass-loading capacity. Moreover, its inherent flexibility, mechanical robustness, and elasticity facilitate the production of wearable biosensors, incorporated into fabrics or dermal patches. These devices can incessantly monitor pH, sweat electrolytes, or metabolites in a noninvasive and real-time manner, especially beneficial for chronic disease management and telemedicine.

**TABLE I.** Comparative Overview of Graphene-Based Biosensor Types.

Biosensor type	Sensing mechanism	Role of graphene	Representative examples	Key advantages
Electrical (FET-based)^10^	Changes in electrical conductance/resistance due to target binding	High carrier mobility, low noise, and large surface area for immobilizing biomolecules	Graphene FET for DNA or protein detection	High sensitivity, label-free detection, and rapid real-time response
Electrochemical^11^	Redox reaction of the analyte at the electrode surface is measured as current or voltage	Enhanced electron transfer, large electroactive area, functionalizable surface	Glucose sensors, dopamine sensors	Low detection limits, rapid response, low-cost, and miniaturizable
Optical^12^	Signal modulation via SPR, fluorescence, Raman scattering, or absorption changes	Strong *π*–*π* interaction for dye loading, fluorescence quenching, SPR enhancement, and high transparency	Graphene-enhanced SERS, FRET sensors	High specificity, multiplexing, compatible with imaging, label-based/label-free
Piezoelectric/Mass-sensitive^13^	Frequency shift in the quartz crystal or the cantilever upon target binding	Large surface area increases mass loading and signal shift	Graphene-modified QCM biosensors	Real-time monitoring, reusable surfaces, label-free detection
Flexible Biosensor^14^	Integration into wearable platforms to monitor biological parameters	Mechanical flexibility, chemical stability, and conductivity support stretchable and conformal sensor design	Sweat-based pH or electrolyte sensors	Noninvasive, continuous monitoring, suitable for telemedicine and home care

To contextualize the efficacy of graphene-based biosensors, it is imperative to compare them with biosensors employing alternative carbon allotropes and novel two-dimensional (2D) materials. Graphene exhibits remarkable electrical conductivity, elevated carrier mobility, and an extensive surface area; nevertheless, alternative carbon materials such as carbon nanotubes (CNTs), carbon black, and carbon quantum dots have also been extensively utilized in biosensing applications. CNTs demonstrate superior electron transport characteristics and elevated aspect ratios; howevere, their one-dimensional architecture restricts surface coverage and may result in heterogeneous sensor interfaces. Graphene offers a flat, uninterrupted sensing platform that exhibits greater compatibility with thin-film devices and surface functionalization. In addition to carbon allotropes, transition metal dichalcogenides (TMDs) like MoS_2_, WS_2_, and WSe_2_ have been extensively investigated for biosensor applications, especially in FET designs. MoS_2_, possessing an intrinsic bandgap of approximately 1.8 eV in monolayer configuration, facilitates elevated on/off ratios in FET-based biosensors and exhibits pronounced photoluminescence, advantageous for optical detection. Nonetheless, MoS_2_ often exhibits inferior carrier mobility compared to graphene and is more vulnerable to environmental deterioration. Likewise, black phosphorus (BP) provides a modifiable bandgap and remarkable sensitivity in optical and electronic biosensors; nevertheless, its chemical instability in ambient settings considerably restricts its practical application. MXenes (e.g., Ti_3_C_2_Tx), a category of two-dimensional transition metal carbides and nitrides, have recently attracted interest due to their elevated capacitance, metallic conductivity, and hydrophilic surfaces. These characteristics render MXenes exceptionally advantageous for electrochemical biosensors. Nonetheless, their production necessitates severe etching processes and yields inconsistent surface terminations, complicating large-scale, reproducible functionalization compared to graphene. Although each 2D material offers distinct benefits, graphene continues to be a prominent contender for biosensing applications owing to its established production methods, environmental resilience, and exceptional electrical characteristics, especially when combined with suitable surface functionalization. A comparative summary is presented in Table II.

**TABLE II.** Comparative summary of the different materials, their properties, advantages, and limitations against graphene.

Material	Key Properties	Advantages in Biosensing	Limitations
Graphene	High conductivity, high surface area, no bandgap	Excellent signal transduction, stable, flexible	No intrinsic selectivity; zero bandgap may limit FET on/off ratio
CNTs	1D, high aspect ratio, high conductivity	Good electron transport, proven functionalization strategies	Limited planar coverage, complex alignment
Carbon Dots	Nanoscale, photoluminescent, biocompatible	Useful for fluorescence biosensing	Lower conductivity, aggregation issues
MoS_2_ (TMD)	2D semiconductor, ∼1.8 eV bandgap	Suitable for FETs, good optical properties	Lower mobility, air sensitivity
Black Phosphorus	Tunable bandgap, anisotropic conductivity	High sensitivity in optoelectronic sensors	Degrades quickly in ambient conditions
MXenes (e.g., Ti_3_C_2_Tx)	Metallic conductivity, hydrophilic, layered structure	High capacitance, strong electrochemical signal amplification	Complex surface chemistry, stability concerns

This review focuses on the recent progress in high-performance biosensing tools by providing broad insights into various fabrication techniques such as mechanical exfoliation, chemical exfoliation, liquid phase exfoliation, electrochemical and microwave-assisted exfoliation, applications in various domains, and characterization techniques of two-dimensional graphene-based materials related to substrates of interest.

## II. GRAPHENE SYNTHESIS AND FABRICATION

### A. Graphene fabrication

Diverse production processes have been established to exploit the distinctive features of graphene for biosensing applications, including electrospinning, freeze-drying, and 3D printing. Electrospinning facilitates the creation of graphene-infused nanofibrous mats characterized by elevated surface area and porosity, which are optimal for biomolecule immobilization and improved analyte interaction. This approach is especially appropriate for developing adaptable, wearable biosensors. Freeze-drying, conversely, enables the creation of porous, three-dimensional graphene-based scaffolds by sublimating solvents at low temperatures, thereby maintaining structural integrity and ensuring excellent surface accessibility. These attributes are advantageous for developing volumetric sensing platforms with swift fluid absorption and diffusion. Three-dimensional printing provides meticulous control over the structure of graphene composites, facilitating the creation of tailored, tiny devices with intricate geometries. It additionally facilitates scalable production and integration with microfluidic systems. Collectively, these production techniques greatly enhance the design flexibility and functional efficacy of graphene-based biosensors in various biological applications. Mechanical exfoliation is an uncomplicated, cost-effective technique that produces high-quality graphene flakes, frequently utilized in fundamental research. Liquid Phase Exfoliation (LPE) entails the dispersion of graphite in solvents, facilitating scale production, while usually yielding worse structural quality. Chemical Vapor Deposition (CVD) yields extensive, high-quality graphene coatings appropriate for electronic applications, albeit necessitating elevated temperatures and intricate transfer procedures. Electrochemical exfoliation provides a more rapid and eco-friendly method for graphene synthesis by applying a voltage across graphite electrodes immersed in an electrolyte. Microwave-assisted exfoliation expedites the exfoliation process by rapid thermal expansion, offering a time-efficient method for graphene synthesis. Laser-induced graphene (LIG) employs laser irradiation to directly transform carbon-rich precursors into porous graphene structures, rendering it particularly appealing for the production of flexible sensors. Each method presents distinct tradeoffs regarding cost, scalability, purity, and applicability; nonetheless, we will examine these techniques more thoroughly to comprehend their processes and practical significance for graphene-based biosensors.

#### 1. Mechanical exfoliation

**FIG. 1.**
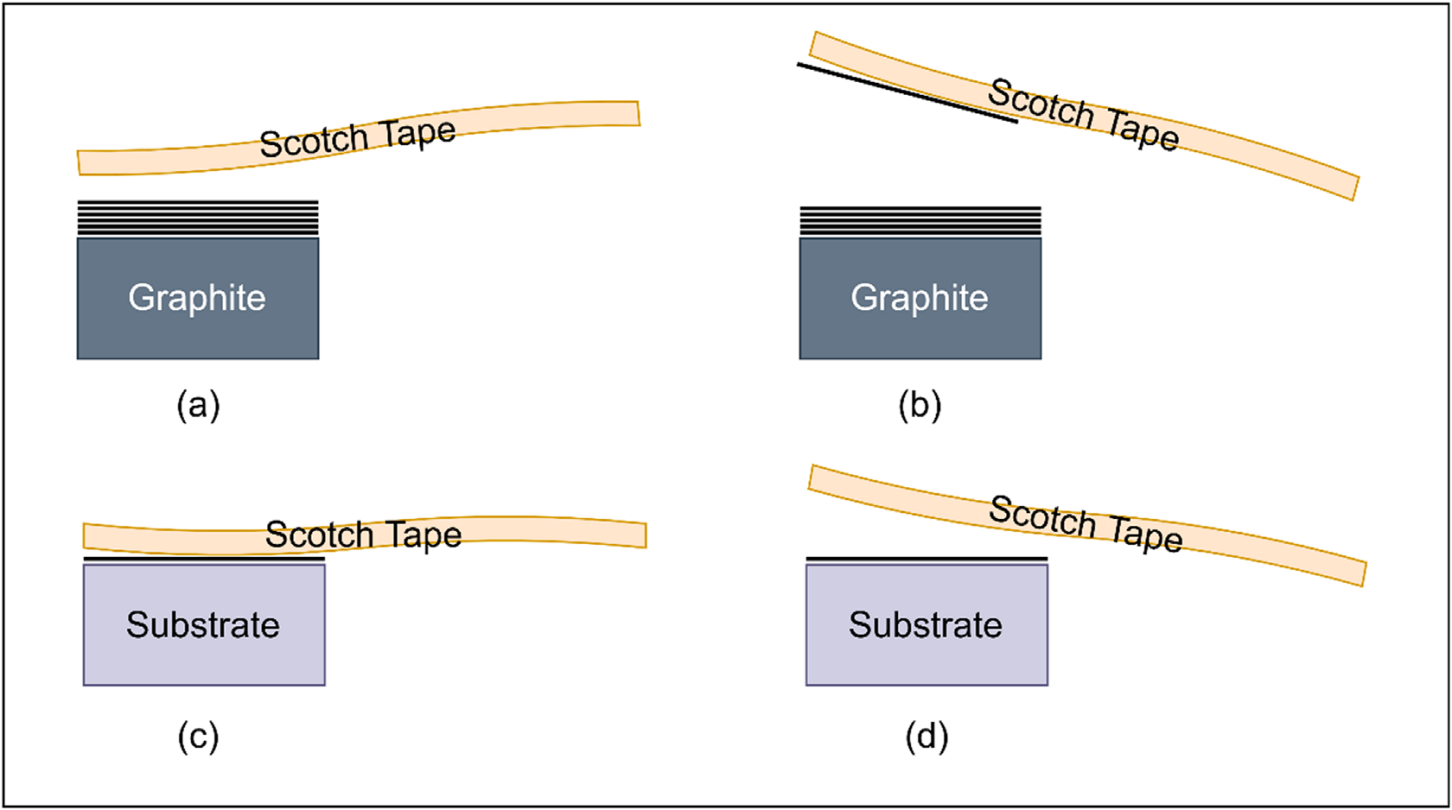
(a) Adhesive tape is pressed onto graphite. (b) Adhesive tape is peeled off when some layers stick to the surface. (c) The tape is pressed onto the target substrate. (d) The tape is peeled off when the layers stick to the target substrate.

Mechanical exfoliation is one of the major techniques for the conversion of graphite to graphene. This method is used to physically separate graphene from bulk graphite. The main concept of this approach is to use a sufficient amount of mechanical force to overcome the weak van der Waals forces existing between the layers of graphene. When the sample of graphite is subjected to enough pressure, the van der Waals forces become ineffective and the layers separate to turn graphene. In this concept, several exfoliation production methodologies have been designed. The first method used to extract graphene from graphite is micromechanical cleavage, or the scotch tape method, presented in Fig. 1. Several graphene layers are created through the process of progressively peeling off the graphite using scotch tape.^15^ Mechanical exfoliation has been widely accepted as it is quite efficient and easy to use (refer Table IV for the summary of key studies on mechanical exfoliation of graphene-based materials, including author names, publication years, and main findings). Subsequent advancements in exfoliation processes included new techniques such as fluid mechanics, sonication, and ball milling. Sonication is another method by which layers of the graphene are separated by subjecting the graphite suspension to ultrasonic waves. It is commonly known that very little fault graphene of high quality is produced by this technique.^16^ Another method, Ball milling, is a mechanical process that converts graphite into graphene by continuously rolling the graphite between the rolls, resulting in scalable graphene suitable for use in industrial environments.^17^ The fluid mechanics techniques that exfoliate graphite into graphene using shear forces in fluids result in high-quality graphene with controllable layer thickness.^18,19^

Saloum *et al.*, stated the process of creating graphene nanosheets from graphite flakes by employing several mechanical exfoliation methods. The technique was recognized because of its high production rate and low cost. TEM and scanning electron microscope (SEM) were used to evaluate the morphological features, and Raman spectra, x-ray diffraction measurements (XRD), and Fourier-transform infrared spectroscopy (FTIR) were used to characterize the structure.^20^ Lugo *et al.*, discussed the mechanical milling and exfoliation of CO_2_ atmospheric synthesis graphene to remove MgO impurity crystals. By burning metallic magnesium in a CO_2_ atmosphere, this environmentally benign method yields MgO and graphene as byproducts. Then, MgCl_2_ and HCl were used to get rid of the MgO. Graphene is a useful reinforcer because of its 125 GPa fracture resistance. The technique showed how to replace the poisonous wastes and dangerous compounds produced by conventional technologies, such as Brodie, Staudenmaier, and Hummers, with a sustainable alternative.^21^ Myroniuk *et al.*, reported the use of polyvinylpyrrolidone (PVP), an organic solvent, in a mechanical exfoliation procedure. A household mixer is used to exfoliate crystalline graphite into graphene in an aqueous solution. SEM revealed the folded form of graphene flakes with a thickness of about 4 nm. Raman scattering revealed high-quality graphene with low defect levels, as evidenced by the low ID/IG ratio of 0.18 and the G band at approximately 1582 cm^−1^. UV-visible spectroscopy was used to confirm that the dispersion contained two-dimensional components.^22^ The goal of the study by Dash *et al.*, was to use naturally occurring high-purity graphite (HPG) to produce reduced graphene oxide (RGO) and graphene oxide (GO) for the aluminum industry. The graphite was exfoliated in a planetary ball mill to remove gaseous reductants and oxidants such as H_2_ and O_2_. TEM, SEM, FTIR, XRD, and Raman spectroscopy were used to characterize the composition, morphology, and structure. To increase the efficiency of aluminum electrolysis, it was suggested that GO and RGO samples, which were found to include four to five layers, be used in electrodes instead of conventional carbon materials.^23^ Fan *et al.*, studied the surface functionalization of graphene oxide (GO) utilizing the bifunctional, trifunctional, and tetrafunctional epoxy monomers (E51, AFG90, and AG80) to improve the dispersion and interfacial properties of epoxy nanocomposites. The study examined the morphological, structural, and chemical characteristics of the GO (epoxy-functionalized) specimens. The interlayer distance increased as the functionality degree of the grafted epoxy monomers grew, with AG80-functionalized GO (AGGO) showing the best exfoliation and dispersion.^24^

Among the many advantages of mechanical exfoliation is its ability to produce high-quality graphene with minimal defects because it is a gentle process. The hybrid surfactants used in the electrochemical-mechanical exfoliation process produced a high yield of 19.68%. The nanosheets' average thickness was roughly 2.4 nm, and their ID/IG ratio of 0.274 showed that they had few defects.^25^ Ball milling with aryl diazonium salts facilitated the industrial-scale production of graphene, where 93% of the platelets had fewer than five layers and low defect levels.^19^ A high-shear-rate microfluidic homogenizer using carbon spheres and water produces high-quality graphene with minimal environmental impact.^26^ *In situ* shear exfoliation within elastomer composites has produced high-performance sensors with remarkable stretchability and sensitivity, which is crucial for biomedical applications.^27^

However, several challenges must be faced while opting for mechanical exfoliation. Even with hybrid surfactants, it is challenging to maintain consistency across batches due to variations in exfoliation conditions.^25^ High shear mixing and probe tip sonication are two examples of energy-intensive mechanical exfoliation techniques that complicate the process and raise production costs.^28^ Some of these methods tend to reduce defects, including the use of polyvinylpyrrolidone (PVP), however, other procedures might introduce undesired elements that compromise the quality and usability of graphene.^29^ These constituents underscore the necessity of continuous optimization to overcome the obstacles linked to mechanical exfoliation.

#### 2. Liquid phase exfoliation (LPE)

**FIG. 2.**
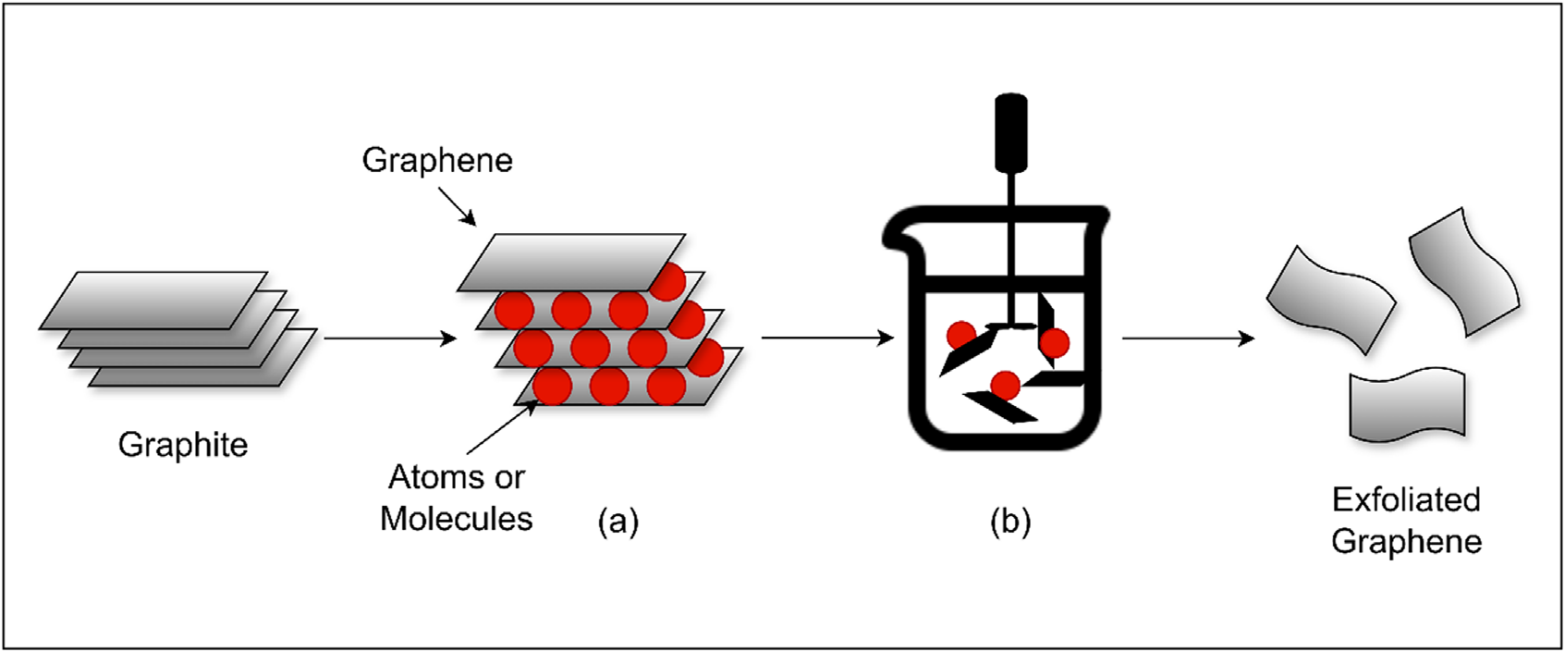
(a) Intercalation process of inserting atoms or molecules in between graphite layers. (b) Sonication process gives a high shear force to Graphite.

Liquid exfoliation or LPE is a method that is used to efficiently produce vast amounts of two-dimensional (2D) materials, including graphene, while balancing cost and quality (refer Table V for the summary of selected works on liquid-phase exfoliation methods for producing graphene and other 2D nanosheets, with details on mechanisms and performance). To separate the layers, the process involves dispersing graphite in a solvent and applying ultrasonic energy as shown in Fig. 2. Due to its relatively easy methodology and capacity to handle massive volumes of data, this method is frequently used in both academic and industrial fields.^30^ There are three steps in the process: first, sound waves are applied to the graphite flakes, creating curved lines that cause fractures to form and solvent to seep in. As a result, thin graphite strips are separated and removed, turning them into graphene.^31^ By optimizing the solvent type and sonication frequency, LPE can produce graphene flakes with minimal defects and excellent quality. Because of this feature, LPE is suitable for many different kinds of applications.^32^ The foundation of liquid-phase exfoliation is the application of ultrasonic waves to a graphite solution, which causes cavitation bubbles. The inward collapse of the bubbles results in strong, localized impacts that disintegrate and separate the graphite into layers of graphene. The contact between the solvent molecules and the graphite layers facilitates the separation and dispersion of graphene flakes, hence improving the process. The efficacy and caliber of the exfoliated graphene are highly dependent on the choice of solvent and ultrasonic settings.^33,34^

Gomez *et al.* state that liquid exfoliation of graphene in polar solvents is the most practical way to create graphene dispersions. The study aimed to optimize variables, including centrifugation conditions, graphite amount, sonication duration, and solvent type. The major findings demonstrated the effectiveness of dimethylformamide (DMF) in exfoliating graphene, producing well-exfoliated graphene nanosheets with few edge defects.^35^ Ma *et al.*, underlined that exfoliating graphene in water-based methods has positive environmental effects. The study examined multiple methods for chemical exfoliation in water, focusing on comprehending the stabilization and exfoliation mechanisms in various aquatic environments and highlighted the importance of using water as an environment-friendly solvent because doing so ensures the process's sustainability and eco-friendliness.^36^ Guler *et al.*, investigated the impact of various liquid media on the amount and quality of graphene produced by direct liquid phase exfoliation of graphite. They found that the quality and efficacy of the exfoliated graphene were significantly improved by the addition of surfactants, such as SDS, to solvents like ethanol and N-methyl-2-pyrrolidone (NMP). As a result, sedimentation times greater than 120 days were reached, and 3–5 layer graphene flakes were produced.^37^ Moosa *et al.*, Examined the efficiency of LPE in producing high-grade graphene. The challenges faced include low monolayer yield and sheet aggregation due to van der Waals forces.^38^ The method of polymer-assisted liquid exfoliation improves graphene dispersion and stability.^39^ The mechanisms of steric repulsion and several forms of interactions (cation-*π*, *π*–*π* stacking, and CH–*π*) combine to form extremely concentrated and stable graphene dispersions.

Studies have indicated that the utilization of polar clean yields a 350% increase in graphene synthesis when compared to conventional solvents, such as *N*-methyl-2-pyrrolidone (NMP). Furthermore, there is a tenfold reduction in defect concentrations.^40^ Graphite can be separated into graphene at a rate of 48 g/h using a plasma spray method, producing high-quality graphene with a high carbon-to-oxygen ratio of 21.2 and few structural defects.^41^

However, LPE also presents several challenges. For example, high-energy sonication, which is necessary for efficient exfoliation, has the potential to cause structural defects in graphene, which would alter its mechanical and electrical properties. For instance, when graphene is divided into layers using solvents like ethanol, the existence of edge-type defects is more apparent.^42^ The method becomes more complex and expensive when post-processing steps, including centrifugation, are used to separate the solvent from the exfoliated graphene. Furthermore, the constraints imposed by the sonication apparatus and the challenges associated with managing large volumes of solvent may make it more difficult to increase graphene production on an industrial scale.^43,44^ To improve LPE for broader industrial applications and ensure the manufacture of high-quality graphene while reducing its environmental impact, these shortcomings must be addressed.

#### 3. Chemical vapor deposition (CVD)

**FIG. 3.**
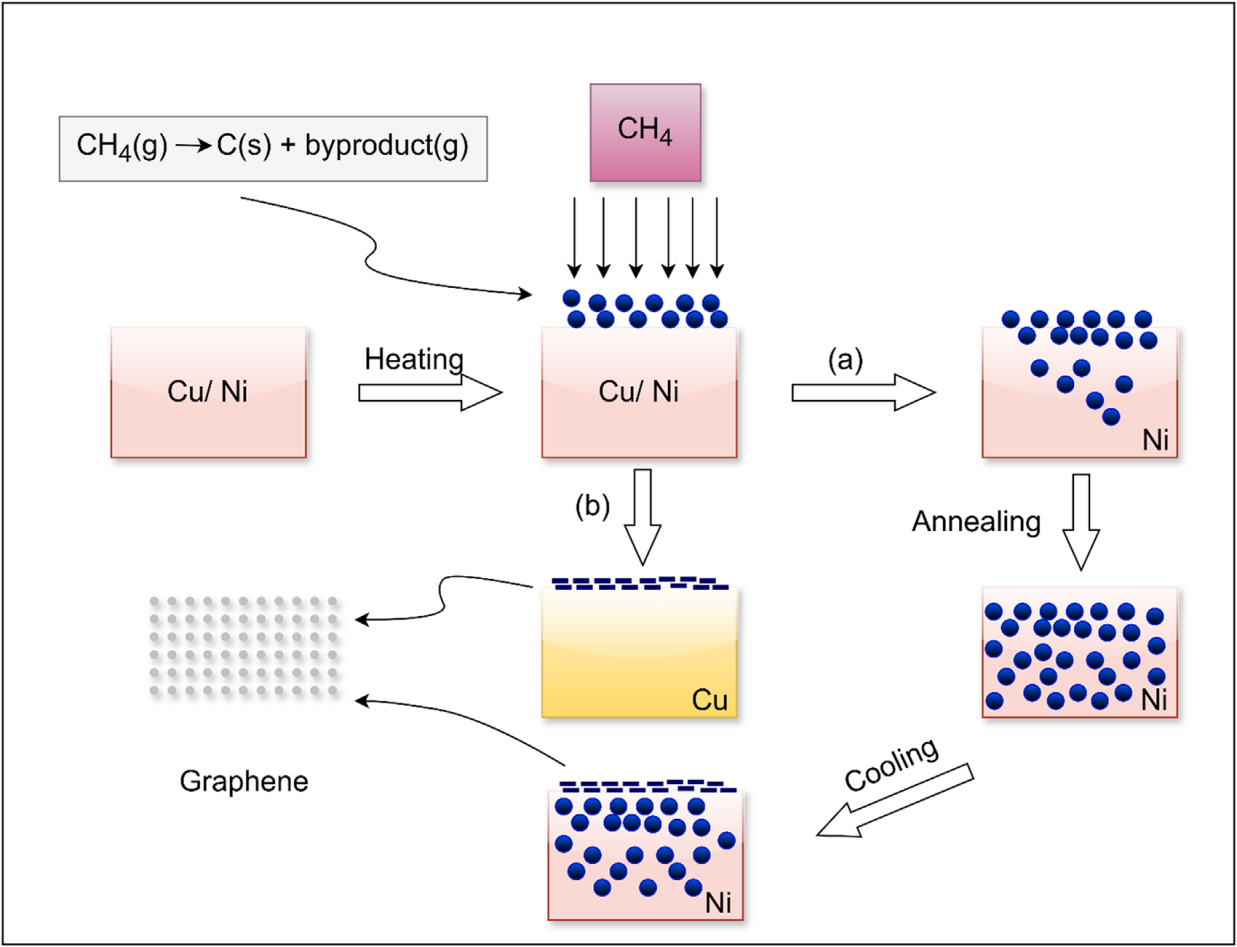
(a) Carbon diffusion on nickel and (b) annealing on copper.

Chemical vapor deposition (CVD) is a widely used technique for producing high-quality, large-surface graphene as shown in Fig. 3. Using this process, carbon-containing gases, such as methane, are broken down on metal substrates at high temperatures (usually around 1000 °C), causing the carbon atoms to organize into graphene layers. The features of graphene, such as its crystallinity, domain size, and number of layers, can be adjusted using CVD's adaptability in regulating growth conditions, such as temperature, pressure, and carbon precursor.^45^ With advancements, CVD can synthesize graphene at temperatures lower than 600 °C, which can save production costs and increase its industrial applications.^46^ The major bottleneck is the difficulty in transferring graphene from metal substrates to surfaces, where problems including surface contamination, doping, and membrane cracking deteriorate the quality.^47^ CVD can be applied to conventional film-like graphene and it has also been modified to synthesize graphene-based fiber materials, which are ideal for electronics and energy storage applications due to their superior mechanical, electrical, and thermal properties.^48^ The core idea behind CVD is the high-temperature chemical reaction of precursor gases, which deposits carbon atoms on a substrate and causes graphene domains to nucleate and develop (refer Table VI for the overview of major advances in chemical vapor deposition (CVD) of graphene, highlighting growth techniques, process parameters, and resulting film properties). So, CVD is used to produce graphene with good crystallinity and uniformity.^49^

In a study by Nandanapalli *et al.*, a green wet-chemical method for depositing graphene layers produced by CVD onto Si/SiO_2_ substrates was described. The work demonstrated the improved quality, purity, and electrical characteristics of the transferred graphene while addressing significant problems with traditional transfer techniques. This method reduces chemical exposure and increases the reliability of the production process, making it a replacement for large-scale applications.^50^ Jia *et al.* studied the clean production of graphene using a cold-wall CVD method. Unlike traditional hot-wall approaches that have surface contamination, this approach effectively suppresses gas-phase reactions, leading to cleaner graphene sheets with improved optical and electrical properties. This technique has ensured the industrial production of graphene, especially for applications requiring highly efficient transparent electrodes.^51^ Zhang *et al.* had given a detailed investigation into the role of hydrogen and oxygen in the development of CVD. The study indicated the ways these gases are used for purifying, creating boundaries, and tuning defects in graphene.^52^ Su *et al.*, through PECVD, prepared graphene platelets for EPDM-based elastomers. The obtained results showed an increase in the dielectric constant of EPDM along with a decrease in its complex viscosity, enhancing the mechanical properties of the material. It illustrated how such advanced composites can be made via PECVD for different industrial applications.^53^ While Wang *et al.* focus on the use of CVD in the production of composites of CNTs/graphene nanoribbons (GNRs), the resultant obtained demonstrates better electrochemical behavior and stability in cycling, probably because of the better structure and conductivity for the GNR/CNT composite. These composites are capable of use in energy storage and membrane capacitive deionization.^54^

With many benefits, CVD has become a successful large-scale graphene synthesis technique. Growing wafer-scale graphene that works with silicon-based electronics and is appropriate for use in integrated circuits, transistors, and sensors is one of its main advantages.^55^ CVD enhances control and repeatability by enabling deterministic synthesis of quasicrystals without the requirement for artificial assembly, such as 30°-twisted bilayer graphene.^56^ Thin-metal films can be used as flexible catalytic substrates in CVD to develop high-quality graphene, increasing its applicability to a wider range of electronic devices by allowing the use of catalytically inactive substrates.^57^ Long-distance spin communication and quantum effects are made possible by CVD-grown graphene, which has also shown impressive improvements in electrical and spin transport performance.^58^ These features are critical for nanoelectronic and spintronic applications. CVD has also been used to create graphene nanoribbons, which demonstrate remarkable gas sensing capabilities.^59^

The process can result in surface contamination and structural imperfections, particularly when using conventional hot-wall systems.^58^ It is necessary to handle graphene carefully during its transfer from the metal substrate to the target surface, as this process frequently produces flaws.^56^ High temperatures are necessary for the growth process, which might restrict scalability and raise manufacturing costs.^57^ Despite these drawbacks, CVD remains one of the most promising methods for large-scale, high-quality graphene synthesis across various applications.

#### 4. Electrochemical exfoliation

**FIG. 4.**
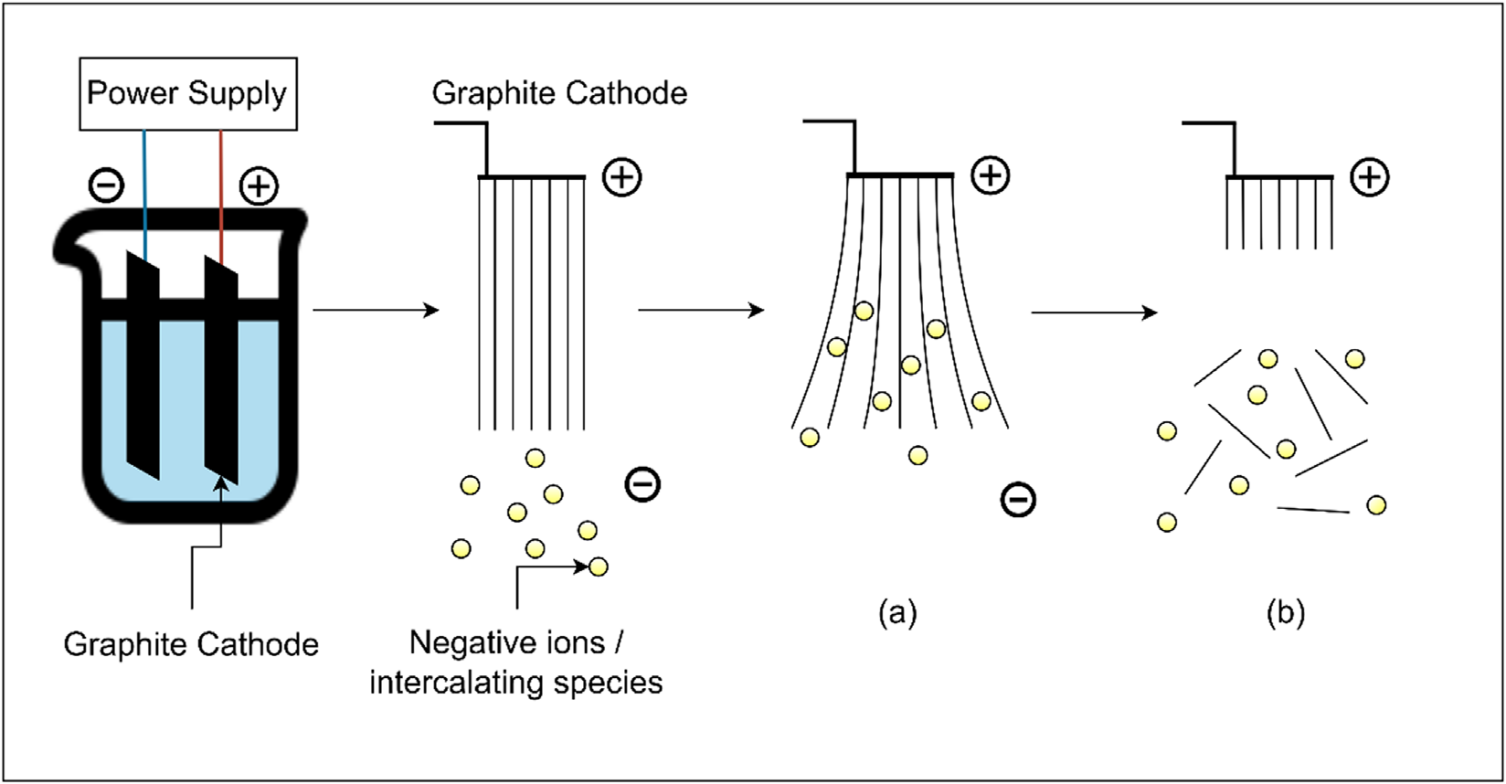
(a) Intercalation of ions into the graphite lattice. (b) Separation of single-layered Graphene.

High-quality graphene can be produced in an economical and ecologically friendly manner by electrochemically exfoliating graphite. This technique involves applying a direct current (DC) voltage to graphite electrodes submerged in an electrolyte solution, causing the ions to intercalate and split the graphene layers as shown in Fig. 4. The yield and quality of graphene that is produced depend on the type of electrolyte that is used, the voltage, and the electrolysis circumstances. For instance, it has been shown that aqueous solutions, such as H_3_SO_4_ and KOH, can be used to create high-quality multilayered graphene with thickness and form.^60^ Several studies have demonstrated that the procedure can be enhanced by modifying variables like voltage and electrolyte content. For example, low-potential DC in electrochemical exfoliation followed by heat treatment was reported to produce few-layered graphene (FLG) with improved crystallinity and surface properties.^61^ Due to relatively low process' cost and scalability, this approach was also suitable for the large-scale synthesis of functionalized graphene.^62^ Recent progress in electrochemical exfoliation has also focused on the use of environmentally benign electrolytes and the incorporation of functionalization processes directly into the exfoliation step itself to tailor the properties of graphene toward application (refer Table VII for the comparative summary of research on electrochemical exfoliation of graphite into graphene, including electrolytes, voltages, and functionalization outcomes).^63^ This technology is adaptable, allowing for the production of graphene-based nanomaterials through simple, one-step procedures like composites of graphene and nanoparticles.^64^

Laudiki *et al.*, stated that graphene oxide (GO) and reduced graphene oxide (rGO) are produced by electrochemically exfoliating pencil graphite at room temperature. The final product was characterized using FTIR, CV, UV-Vis, XRD, and other physical and electrochemical techniques. The results demonstrated the material's consistent dispersion at controlled oxidation levels and demonstrated the potential of GO/rGO for sensor applications.^65^ Ji *et al.*, examined the possibility of combining carbon black (CB) and reduced electrochemically exfoliated graphene oxide (rEGO) to boost the efficiency of platinum (Pt) catalysts in hydrogen fuel cells.^66^ The hybrid material retained 71% of its original electrochemical surface area and showed enhanced ORR activity after 30 000 cycles under stress tests. The authors concluded that rEGO/CB hybrid support may have an enormous impact on fuel cells in terms of durability and performance. Danial *et al.* provided a review on the advantages that electrochemical exfoliation offers in graphene and GQDs preparation. Advantages implied here using this strategy compared to conventional methods include simplicity, safety, and effectiveness. The authors also identified that, for such graphene production in an agglomeration-free and dispersible manner, there are still many challenges to overcome regarding the wide-scale applications of graphene.^67^ Li *et al.* focused on graphene-based hybrid materials fabricated by electrochemical exfoliation in the review. This work underlined the mild conditions, short processing time, and ease of this process to obtain GHMs with a wide variety of applicational fields: electronics and energy storage. The study also explored variables affecting hybridization and suggested some lines of inquiry that future research could take in an effort to improve both the quality and scalability of GHMs.^68^ Lee *et al.* studied the role that anions play during electrochemical exfoliation of graphite into graphene. It showed that sulfate anions are effective exfoliators in comparison with other anions, like nitrate or chloride, due to the peculiar dealings that happen with graphite layers. The conclusions of this work provided evidence of how crucial the composition of electrolyte is in order to maximize exfoliation and get higher concentrations of quality graphene.^69^

The simplicity, affordability, and environmental friendliness of electrochemical exfoliation make it a potential technique for graphene synthesis. One of its primary advantages is the ability to produce high-quality graphene oxide (GO) and reduced graphene oxide (rGO) with tunable properties by adjusting parameters such as voltage and electrolyte composition.^65^ The technique is adaptable, enabling the exfoliation of different graphite sources, such as waste materials like dry cell batteries and naturally occurring graphite flakes, enabling scalable and affordable manufacturing.^70,71^ Additionally, electrochemical exfoliation makes it easier to add dopants, such as chlorine, to improve the performance of materials based on graphene, for applications like micro-supercapacitors that require great mechanical flexibility and energy density.^72^ Ultrasonic assistance is one technology that can be used to improve the process and increase the scalability and efficiency of graphene manufacturing for large-scale applications.^73^ Since electrochemically exfoliated graphene exhibits high conductivity and stability, it is a potential anode material for lithium-ion batteries. It also has a high capacity and strong cycle stability.^74^

Despite these advantages, electrochemical exfoliation presents several disadvantages. For example, the probable inability to easily control the degree of oxidation and achieve homogeneity in the final product, which represents a source of variation in quality and performance, is a major problem.^70,71^ The environmental problems and hazards of handling due to the use of strong acids or particular electrolytes are also foreseen, even though substantial improvements have been made toward the adoption of less unsafe reagents. This method is very good overall, but more research is needed to determine its parameters so that it can satisfy difficulties related to environmental effects and product uniformity.

#### 5. Microwave-assisted exfoliation

**FIG. 5.**
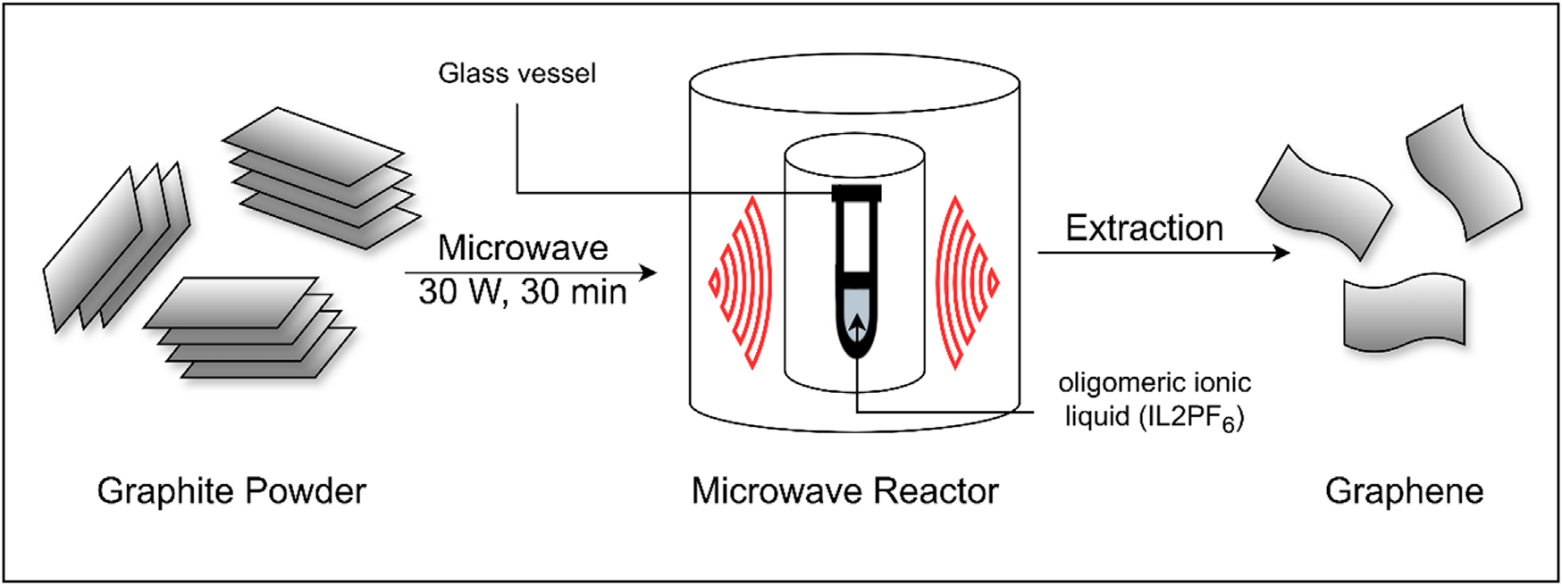
Microwave-assisted exfoliation of Graphene in oligomeric ionic liquid.

Microwave-assisted exfoliation of graphene has emerged as an efficient, scalable, and eco-friendly method for producing high-quality graphene, as shown in Fig. 5. The idea behind this method is that microwave radiation interacts with the *π*-electrons in graphene to provide rapid exfoliation. Without the use of hazardous chemicals or long processing time, as is the case with traditional procedures, this method exfoliates and reduces graphene oxide (GO) or graphite into few-layered graphene faster.^75,76^ Graphite or graphene oxide is exposed to microwave radiation, which causes layers to expand and exfoliate. The efficient reduction of graphene oxide (GO) is facilitated by the volumetric heating provided by microwaves, which also increases the interlayer gap and removes oxygen functional groups, improving the conductivity and structural properties of the resulting graphene.^77^ Various solvents have also been used in conjunction with the procedure to enhance the exfoliation process. Solvents having dipole moments between 2 and 4 Debye have been shown to significantly increase the electrical conductivity of the resulting graphene.^78^ Microwave-assisted exfoliation can be employed in large-scale production due to its advantages, which include quicker processing times, reduced energy use, and less environmental effect (refer to Table VIII for the key publications on microwave-assisted exfoliation of graphite for graphene synthesis, detailing solvents, power settings, and product quality.).

Microwave-reduced graphene oxide (MWrGO) exhibits enhanced structural stability and electrochemical properties, and the technology has also been used to produce graphene for innovative applications, such as high-performance anode materials in lithium-ion batteries.^79^ This procedure provides a feasible route to the commercial production of graphene with certain properties for a variety of technological applications.

Kumar *et al.* studied microwave-assisted fabrication methods. The study demonstrated how microwave processing, utilizing a variety of materials such as metal oxides, carbon nanotubes, and polymers, improves the electrodes' electrochemical performance, to quickly produce high-quality graphene-based composites. According to the study, microwave irradiation is a potential method for energy storage applications because it can both exfoliate and decrease graphene oxide at the same time.^80^ Recent developments in microwave-assisted techniques for creating two-dimensional materials (2DMs) studied by Wang *et al.* The study found that the structural and catalytic properties of 2DM-based materials can be enhanced by microwave processing, increasing their applicability in catalysis applications.^81^ Skorupska *et al.* conducted a study on nitrogen-doped graphene foam produced by microwave-assisted exfoliation. The project's objective was to develop electrochemically active, ecologically acceptable energy storage materials. Strong electrochemical activity was demonstrated by nitrogen-doped graphene produced by microwave techniques, particularly for oxygen reduction reactions (ORR). The material showed better ORR activity as compared to that of traditional platinum-based catalysts, which makes it a sustainable alternative for energy-related applications.^82^ A microwave method backed by ultrasound was presented by Yang *et al.* for the secondary exfoliation of electrolytic graphene oxide. The outcomes created a few-layer graphene with a thickness of about 1 nm and a C/O ratio of 15.2. The industrial-scale manufacturing of high-quality graphene with low defect levels has shown significant potential when ultrasonic and microwave methods are combined.^83^ A study on the use of microwave-assisted intercalation expansion by Knuth *et al.* found that heating the material at 800 °C is the most effective way to produce high-quality graphene nanosheets. Cyclic voltammetry (CV) and electrochemical impedance spectroscopy (EIS) analyzed the electrochemical characteristics of these nanosheets, which had a thickness of about 1 nm.^84^

Microwave-assisted graphene synthesis techniques are a scalable and effective way to create high-quality graphene-based materials. According to Kumar *et al.*, holey graphene nanosheets (HGNS) with enhanced electrical conductivity and pore size distribution—suitable for energy storage applications—can be created by microwave-assisted expansion.^85^ Chen *et al.* demonstrated that microwave irradiation can simultaneously exfoliate and decrease graphene oxide (GO) because of its quick and consistent heating, improving the electrochemical performance of graphene materials in supercapacitors.^86^ Zhang *et al.*demonstrated how microwave-assisted methods can incorporate nanoparticles, including cobalt oxide (Co_3_O_4_/CoO), into reduced graphene oxide (rGO), enhancing the material's stability and electrochemical characteristics for use in supercapacitors.^87^ Additionally, Kumar *et al.* noted that microwave treatments can increase the surface area and porosity of conducting polymers based on graphene, which is crucial for increasing energy storage capacity. However, the studies also pointed out that because polymeric materials like polyaniline and polypyrrole are structurally sensitive, microwave treatment can be difficult.^88^ Further expanding its range of applications, Chen *et al.* discovered that microwave-assisted exfoliation of graphene oxide (GO) in conjunction with TiO_2_ nanoparticles can cause GO sheets to rip into smaller graphene quantum dots.^89^ Although microwave-assisted synthesis has many benefits, its applicability to all material systems is limited by the possibility of inconsistent material properties, such as partial reduction or fragmentation based on the irradiation power, and difficulties in controlling the process when polymer composites are involved.

#### 6. Laser-induced graphene (LIG)

LIG is an innovative and widely embraced method for the quick, economical, and scalable production of graphene, especially applicable in biosensor manufacturing. This technology, initially devised by Tour *et al.* in 2014, employs a laser scribing technique to directly transform carbon-rich substrates, such as polyimide, wood, paper, or food waste, into porous graphene films under ambient circumstances.^90^ The primary advantage of LIG is its maskless, chemical-free, and transfer-free characteristics, which considerably reduce the obstacles to integrating graphene devices compared to other production methods such as chemical vapor deposition (CVD) or liquid-phase exfoliation. LIG is formed via localized photothermal heating from a CO_2_ or fiber laser, which disrupts C–H, C–O, and other non-sp^2^ bonds in the precursor material, resulting in interconnected networks of graphene-like carbon atoms. The resultant LIG is not monolayer graphene; instead, it is a porous, turbostratic graphene foam distinguished by a significant level of disorder, edge defects, and three-dimensional shape. The structural characteristics, albeit distinct from pure graphene, enhance biosensing by augmenting the electrochemically active surface area and offering numerous anchoring sites for bioreceptor immobilization.^91^ LIG exhibits significant adaptability to various carbonaceous precursors, rendering it appropriate for versatile, biodegradable, and economical substrates. In addition to polyimide, recent research has shown effective LIG production on paper, cellulose, cork, lignin, food waste (such as bread or coconut shells), and silk fibroin.^92^ This extensive substrate compatibility facilitates sustainable, and environmentally friendly sensor design and broadens the spectrum of applicable biosensing devices to encompass wearables, transient electronics, and environmental monitors. Additionally, LIG can be shaped *in situ* with great spatial precision, enabling researchers to directly fabricate intricate microelectrode arrays or fluidic channels without the need for photolithography.^93^ This has facilitated advancements in lab-on-a-chip biosensors, integrated microfluidic systems, and entirely printed diagnostic platforms.

LIG-based electrodes in biomedical biosensing have been effectively functionalized with enzymes, aptamers, and antibodies for the detection of glucose, uric acid, dopamine, and lactate. These sensors have superior performance for sensitivity, linear range, and stability, frequently equaling or exceeding that of CVD-graphene-based devices. The porous structure of LIG enables rapid analyte diffusion and efficient bioreceptor loading, while its high conductivity enhances signal transmission.^94^ A significant application is the creation of skin-mounted LIG biosensors for sweat analysis, facilitating real-time and noninvasive health monitoring. Lin *et al.* presented a flexible LIG-based glucose biosensor that exhibited signal stability during mechanical deformation, underscoring the potential of LIG for next-generation wearable diagnostics. In addition to biomedical applications, LIG has demonstrated potential in environmental biosensing,^95^ particularly for the detection of heavy metal ions (e.g., Pb^2+^, Hg^2+^), pesticide residues, and endocrine disruptors in water samples. The use of chelating agents or metal nanoparticles (such as gold or bismuth) enables LIG sensors to attain low detection limits while preserving great selectivity. The porous structure facilitates concurrent multi-analyte detection, paving the way for multiplexed environmental monitoring systems. A primary advantage of LIG is its industrial scalability. The fabrication process is suitable for roll-to-roll manufacturing, robotic laser writing, and integration with flexible or disposable substrates. LIG may be synthesized at ambient pressure without the need for catalysts, cleanrooms, or vacuum systems, rendering it an optimal platform for economical, mass-producible biosensors, particularly in resource-constrained environments. Moreover, the interoperability with additive manufacturing and 3D-printing technologies has facilitated the creation of LIG-based microfluidic and electrochemical systems, which are advantageous for quick prototyping and on-site testing. A comparison between the different fabrication methods has been shown in Table III.

**TABLE III.** Comparison between different fabrication methods based on Yield, Defect Density, Scalability, and Suitability.

Method	Yield	Defect Density	Scalability	Suitability for Biosensors
Mechanical Exfoliation	Low	Very Low (high-quality graphene)	Low	Suitable for research-scale sensors; poor for large-scale use
Liquid Phase Exfoliation (LPE)	Moderate to High	Moderate (depends on sonication control)	Moderate	Good, tunable flake size and functionalization possible
Chemical Vapor Deposition (CVD)	Moderate	Very Low (crystalline graphene)	High (with automation)	Excellent, high uniformity and conductivity
Electrochemical Exfoliation	High	Low to Moderate	High	Excellent, scalable, cost-effective, and green
Microwave-Assisted Exfoliation	High	Moderate	Moderate	Good, rapid, green, but optimization needed for uniformity

## III. APPLICATIONS OF GRAPHENE-BASED BIO-SENSORS

### A. Biomedical sensors

**FIG. 6.**
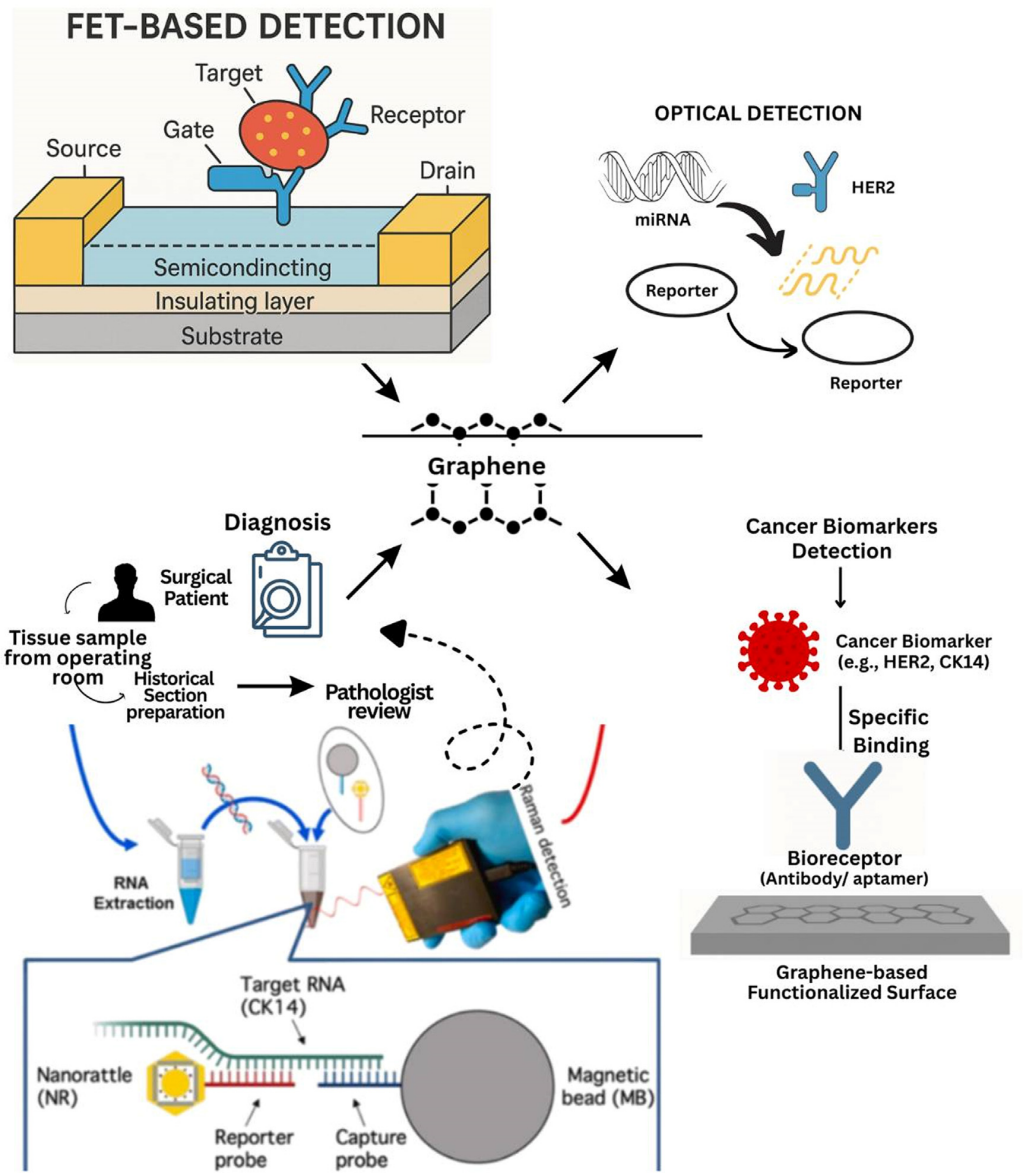
Biomedical application of graphene sensor.

Graphene-based biosensors show developments in the field of cancer detection as shown in Fig. 6. Due to its unique properties, a graphene sheet is highly suitable for early diagnosis, including biomarker analyses, because it shows high surface area, good electrical conductivity, mechanical strength, and biocompatibility (refer Table IX for the applications of graphene-based materials in cancer diagnostics, summarizing biosensor designs, targets, and analytical performance metrics.). These features allow very sensitive, accurate, and fast diagnostic responses from graphene-based biosensors, electrochemical and optical biosensors have shown results in enhancing the detection of key cancer biomarkers, i.e., carcinoembryonic antigen (CEA), prostate-specific antigen (PSA), and cancer-related exosomes. FETs and fluorescence resonance energy transfer (FRET) are used to enhance sensitivity and selectivity. These biosensors are suitable for diagnostics and cost-effective clinical deployment.^96^ Graphene-based FET biosensors have significantly overpowered conventional diagnostic methods, and their ultra-sensitive, label-free detection capabilities have attracted a great deal of attention. These sensors offer high accuracy in identifying cancer biomarkers at low concentrations. FET biosensors have demonstrated the ability to detect PSA at femtomolar levels and differentiate between cancerous and non-cancerous cells with precision, demonstrating their potential in cancer diagnostics.^97^ The diagnosis of breast cancer became easy with these FET biosensors. Aptamers are single-stranded DNA or RNA molecules that bind to targets, and their introduction improves the stability and adhesion of receptors to graphene surfaces. Allowing detection limits as low as 20 aM for miRNA and 0.6 fM for HER2 proteins, which offer diagnostics faster and more precisely than previous methods, this study depicts the potential of FET biosensors in revolutionizing diagnostics for cancer.^98^ Graphene-based electrochemical biosensors have also shown promising results in the early detection of breast cancer. These sensors detect biomarkers related to breast cancer, such as CA 15–3 and miRNA-21, with high accuracy. Among such advanced modification approaches that enhance bioreceptor immobilization and increase sensor performances, the incorporation of gold nanoparticles has resulted in higher sensitivities. The low detection limits, attained in the femtomolar range, show the potential of these biosensors for early intervention, which is crucial for improving the outcomes of breast cancer treatment. The combination of all these advanced biosensing techniques has increased the accuracy and efficacy of early diagnosis manifold in breast cancer.^99^ Graphene-based biosensors are not restricted to electrochemical and FET techniques only. They have also been effectively used in combination with several detection methods: fluorescence, surface plasmon resonance (SPR), and surface-enhanced Raman scattering. Fluorescent biosensors use graphene quantum dots (GQD) to detect cancer biomarkers. Such diagnostics, performed in a noninvasive real-time manner by these biosensors, are useful in cancer monitoring and detection. Furthermore, GO-aptamer systems and rGO-decorated electrodes significantly improved biomarker identification by improving low detection limits and performing efficient analysis of complex biological samples to allow more accurate and accessible diagnostics of cancer.^100^

Another important area of application forgraphene-based biosensors is the detection of lung cancer. Graphene biosensors are designed to detect specific biomarkers for tumors, including EGFR, TP53, KRAS, neuron-specific enolase (NSE), CYFRA 21–1, and carcinoembryonic antigen (CEA), which are important in the diagnosis and follow-up of NSCLC diseases. These sensors depend on the use of electrochemical transduction that offers very high sensitivity and specificity. These devices function better and are more stable because graphene facilitates functionalization and enhances charge transport. With the advancement of advanced transducer technologies, including mass-based and optical systems, the application of graphene-based biosensors has expanded in lung cancer diagnostics, allowing more practical, portable, and real-time applications able to considerably improve patient outcomes.^101^ GO-based electrochemical biosensors are used in the detection of tumor cells, proteins, and microRNAs. FET-based GO biosensors have been employed for monitoring prostate cancer. Furthermore, GO can be used for signal amplification with the ability to conduct ultra-sensitive detection. Paper-based devices also have great potential in the detection of breast cancer indicators since they offer a portable and reasonably priced option for diagnostics of cancer.^102^ The development of CNT/graphene hybrid biosensors has significantly enhanced the capability in cancer detection. Such biosensors are capable of detecting a variety of cancer biomarkers like proteins, nucleic acids, inflammatory markers, IL-6, PSA, IGF-1, and miRNA. The functionalization of carbon nanotubes and graphene allows the label-free detection of these biomarkers. These advantages are very important for the correct and timely diagnosis of cancer, improving both patient satisfaction and treatment outcomes.^103^ Graphene-based biosensors are used to monitor drug response, detect biomarkers such as HER2, CA125, and PSA, various CTCs in the blood, and monitor the efficacy of treatments against cancers. This contributes to a significant impact on cancer care, as the dissemination of cancer metastases will be possible to evaluate and build treatment models suitable for specific patients.^104^ An electrochemical biosensor capable of detecting the prostate cancer marker miRNA-21 has a low detection limit of as little as 3 fM, enabling real-time, label-free diagnosis. Covalent immobilization of probe DNA on graphene surfaces significantly enhances their stability and repeatability, hence ensuring better consistency and reliability during clinical performance.^105^ Though clinical verification of these graphene biosensors is still in its infancy stages, there is limited human trial evidence that is confined largely to *in vitro* and small-scale *in vivo* trials. Sensitivities of 85%–98% and specificities of 80%–95% for different cancer biomarkers have been reported but need verification across different population groups and clinical settings. Hindrances toward commercialization of the device include standardization of the device, reproducibility of the fabrication of the sensors, interfacing with the healthcare infrastructure, and adherence to standards such as FDA and CE marks. Both cost-effectiveness and scalability become a concern with the shift from the lab prototypes toward commercially viable diagnostic technologies. These factors have to be resolved in a way that enables the graphene biosensors to transition from experimental technologies into clinically reliable diagnostic platforms.

### B. Food and pharmaceutical quality control sensors

**FIG. 7.**
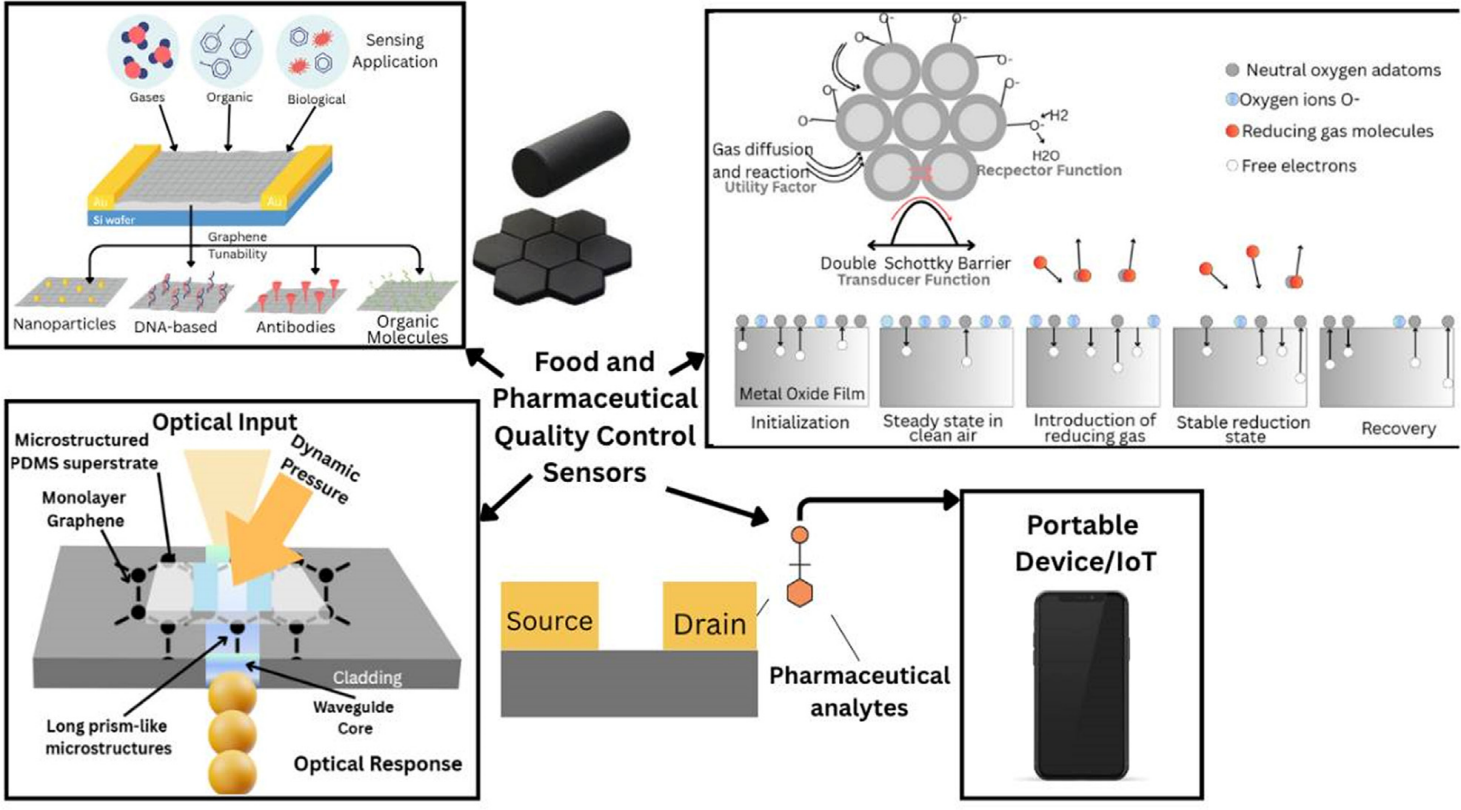
Food and pharmaceutical quality control application of graphene sensors.

Carbon nanostructures, such as graphene and CNTs, are highly promising constituents of biosensor transducers because they are characterized by high sensitivity and broad perspectives of multi-modal applications in food analysis, as shown in Fig. 7. The large surface area and catalytic activity of these nanostructures improve the extraction of the analytes as well as electrode performance in the biosensors (refer Table X for the Graphene-based biosensors and nanosensors for food safety and contaminant detection, summarizing analytes, transduction principles, and sensitivities and refer Table XI for the graphene-based biosensor platforms in pharmaceutical and therapeutic monitoring, detailing analytes, sensing strategies, and device performance.). Their stability under extreme conditions enables their applications in various biosensing platforms, such as electrochemical, optical, chemo-resistive, and field-effect transistor-based techniques toward the rapid detection of food adulterations. It presents the recent development in the capabilities and potential of portable in-field analytical devices.^106^ Abdelbasset *et al.* studied the broad applications of biosensors, with great emphasis on their applications within food, environment, and health. Biosensors combine biochemistry and molecular biology with telecommunication engineering to achieve high specificity and sensitivity for the detection of biological molecules and contaminants. The integration of advanced technologies such as radio, optical, electromagnetic waves, and micro-strip antennas in biosensors leads to their increasing application in food safety and quality analysis.^107^ Peña-Bahamonde *et al.* focused on graphene's rare physical and chemical properties, and therefore, perfect suitability, for biosensor applications. It covers recent advances in the field of biomolecule immobilization, such as antibodies and enzymes, with graphene-based sensors for improving sensitivity and selectivity during the analysis of food. The simplicity and speedier response of electrochemical detection methodologies are highlighted, especially rendering graphene biosensors useful in medical and food safety applications.^108^ Sundramoorthy *et al.* have pointed out that there is a need to develop innovative solutions for food safety because of globalization in the chain of food supply. Graphene shows very good suitability for electrochemical and optical sensing platforms due to its high conductivity and possibility of functionalization. The study also looks into the technologies of smart food packaging such as radio frequency identification (RFID) and freshness indicators, improving monitoring and quality assurance options within the food supply chain.^109^ Hou *et al.*, studied the necessity of rapid and reliable detection systems for monitoring food contaminants. Among the different techniques, electrochemical sensor techniques are gaining major relevance in food analysis due to their high sensitivity of the results. The authors discussed advances and challenges toward the development of new electrode materials leading to improved performances in food analysis, while trying to meet the urgent need for reliable and multi-analyte detection systems in food safety.^110^ Taniselass *et al.* presented an overview of graphene-based materials, such as graphene oxide and reduced graphene oxide and discussed their applications in the fields of food safety, environmental monitoring, and biosensing. The study describes recent advances in graphene-based electrochemical biosensors for detecting markers of specific non-communicable diseases with high selectivity and sensitivity. Functionalization of the graphene surface is conveniently performed to attach biomolecules, rendering such sensors effective for a number of different applications ranging from food control to health monitoring.^111^ Li *et al.* identified 2D materials like graphene, MXenes, and transition metal dichalcogenides applied to biosensors in food safety. In addition to the explanation of synthesis and surface chemistry, the study discussed strategies of functionalization that enhance the detection of hazardous substances like nitrites, heavy metals, and antibiotics in foods and focused on the extent of biosensors that are into monitoring human health markers and food quality using 2D materials for supporting their usage in foods' safety technologies in the future.^112^ Neethirajan *et al.*, biosensors targeted allergen detection in food, addressing the rising concerns related to food allergy challenges. The various types of sensors of allergens are characterized according to their mechanisms, including optical, electromechanical, and electrochemical-novel nanosensors and cell-based assays. The challenges in allergen monitoring and the biosensors that could help avoid any accidental exposure to allergens were discussed. It has also highlighted the need for the food industry to have some kind of standardized determined levels for effective detection.^113^ Shahriari *et al.*, discussed graphene and graphene oxide modification advances for biosensing applications to address issues with graphene, such as inertness and issues relating to conductivity, and discussed functionalization techniques that give rise to efficient biomolecule–biomolecule interaction and immobilization—a key theme for food safety and environmental monitoring. Graphene nanomaterials have been pinpointed to act as the basis of developing stable biosensors which allow for signal response improvement and biocompatibility.^114^ Lv *et al.* highlighted the role of nanomaterials-based biosensors applied in food safety, offering rapid, sensitive, and portable detection of contaminants like pathogens, heavy metals, and illegal additives.^115^ Most of the future directions will bridge the gap between food science and nanotechnology in the advancement of biosensors for food safety, thereby allowing for the wider acceptance of nanomaterials-based approaches for contaminant detection within food.

Redzepi *et al.* explored the role of graphene and other carbon nanomaterials in the pharmaceutical field. The use of graphene in advanced diagnostics, drug release, and tissue engineering shows how it allows the possibility of early detection of disease while at the same time enhancing patient care by precision in biosensing.^116^ Sharma *et al.* discussed the excellent electrical and physical properties of graphene, its interesting uses in biomedicine and pharmaceuticals, and the contribution of graphene-based biosensors to the diagnosis and early detection of pathogens, further underlining the development of such sensors for wide ICT detection of biomolecules. It is noted that graphene has versatile health monitoring, including drug delivery and gene transfer, because of its high sensitivity, selectivity, and surface adaptability.^117^ Savita *et al.* focused on the synthesis and resultant toxicity of graphene nanomaterials (GNMs), with a focus on their biomedical applicability. The study discussed a few synthesis techniques and *in vitro*/*in vivo* toxicity studies regarding GNMs. The toxic residues, which might have been incorporated during certain synthesis processes, have the potential to challenge safe clinical use. Insight into information on bioavailability and cytotoxicity regarding GNMs shows the requirements of safer synthesis approaches when pharmaceutical applications are concerned.^118^

Recent publications have highlighted graphene nanomaterials as implantable biosensors and drug delivery platforms, a focus area in bioengineering. Kulakova *et al.* discussed the novel physicochemical properties of graphene nanomaterials (GNMs) that facilitate their employment as targeted drug delivery carriers with tunable surface chemistry and high drug capacity.^119,120^ Challenges in toxicity and material integration into living systems were, however, noted by the authors as significant impediments toward clinical translation. Hemdan *et al.* proceeded with the exploration of the coupling of nanotechnology with biosensor miniaturization for real-time monitoring of therapeutics, such as for drug therapy monitoring in wearables or implant contexts. Their review identified advancements in the sensitivity and specificity of the data as well as technical issues such as reproducibility and data integrity.^125^ Sabah Ahmed *et al.* presented a graphene oxide-polyaniline biosensor that detects bilirubin with the potential of such systems providing continuous biochemistry feedback pertinent to drug metabolism and liver health when minimized and implanted *in vivo*.^121^ EKSIN *et al.* discussed electrochemical drug–DNA interaction analysis using fullerene-enriched disposable electrochemical sensors with promises of providing body-interior pharmacokinetics as well as drug safety profiles.^122^ These contributions jointly reflect the increased applicability of graphene-enabled implantable biosensing platforms in personalized drug delivery and biomedical monitoring, a central focus of bioengineering research. Kadhim *et al.* discussed the use of graphene oxide-based nano-biosensors for early detection of lung cancer with particular attention to DNA-GO nanohybrids. The paper showcases how the sensor can identify deletion mutations associated with lung cancer with high sensitivity using fluorescence spectrometry methods. This underlines the potentiality of graphene oxide to create low-cost, rapid diagnostic tools that will answer clinical demands because there is an increasing need for early methods of diagnosing cancers.^123^ Laghlimi *et al.* underlined the most recent improvements of sensors by graphene, fullerenes, and carbon nanotubes, improving their sensitivity and speeding the analytical process. These studies confirm the interest of the pharmaceutical industry in developing sensors as a tool for the analysis of drugs or biological samples at low cost in a very short time.^124^ Hemdan *et al.* provided an overview of biosensor technologies for therapeutic drug monitoring and disease biomarker detection. This assesment includes recent innovations in integrating nanotechnology, wearable devices, and miniaturization, which enhance biosensors' sensitivity and specificity. The study discusses data security, reproducibility, and other challenges in biosensing, proposing solutions to these issues, which are essential for improving healthcare outcomes in pharmaceutical applications.^125^

### C. Wearable and point-of-care sensors

**FIG. 8.**
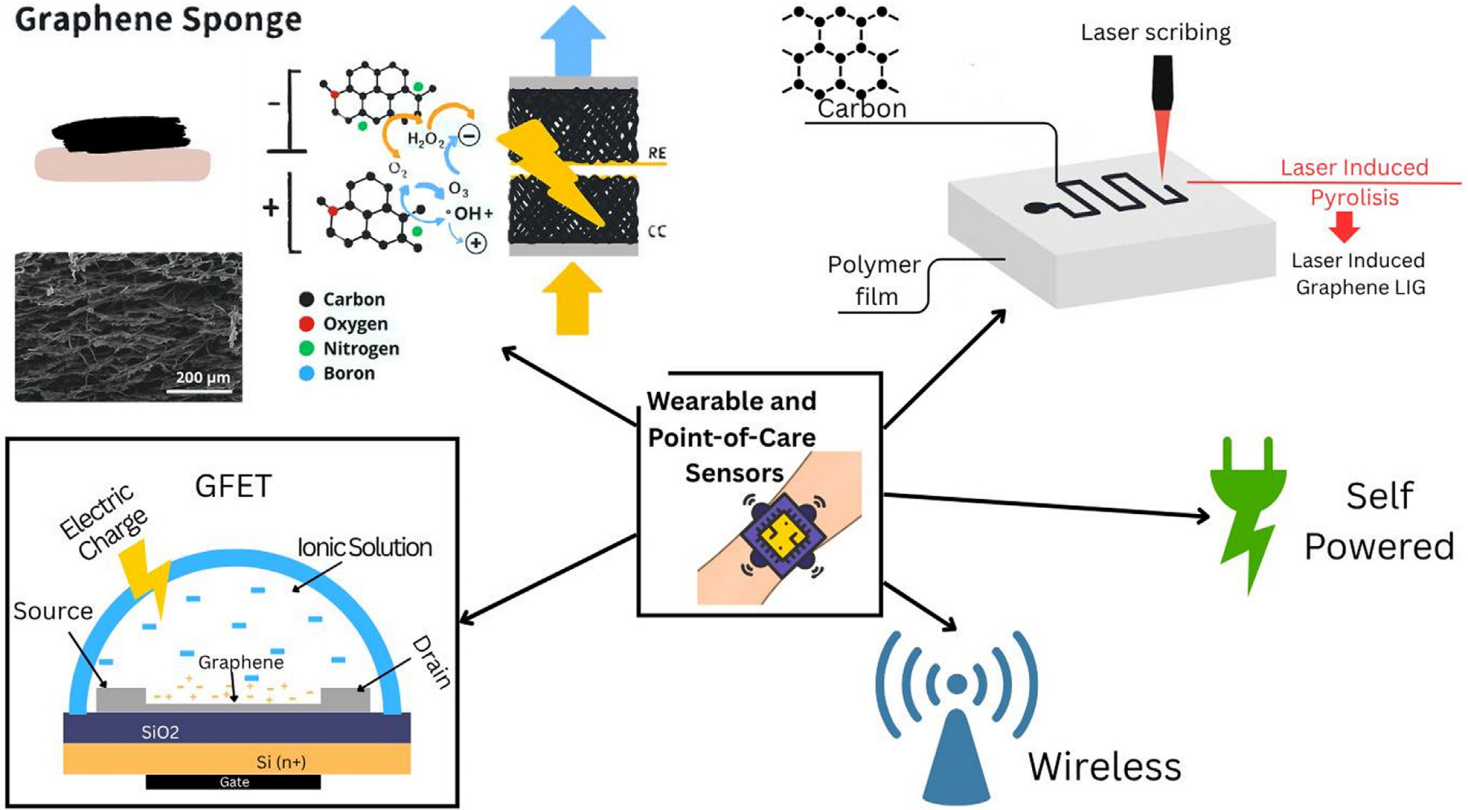
Application of wearable and point-of-care graphene sensors.

The physical, chemical, and electrical properties of graphene make it perfect for developing wearable devices intended for healthcare and personalized health tracking, as shown in Fig. 8. Its high conductivity, flexibility in nature, mechanical strength, and biocompatibility allow the designing of sensitive, noninvasive, and portable sensors (refer Table XII for the development of wearable graphene devices for biochemical and physiological monitoring, including sensor configurations and detection limits.). Such sensors are imperative in real-time monitoring of biomolecules, gases, and other health indicators, leading to preventive and personalized healthcare solutions. Recent fabrication methods represent graphene field-effect transistor-based devices as wearable biosensors. Hu *et al.*,^126^ studied the flexible GFETs, graphene has its biocompatibility, adaptability, and mechanical flexibility for an ideal choice for wearable applications used to detect biomolecules such as proteins, glucose, and ions which shows the non-encapsulated and external gate arrangements that enhance the performance of these biosensors, overcoming their challenges with environmental contamination and extending possibilities for continuous health monitoring. A graphene sponge-based flexible enzymatic biosensor has been developed capable of noninvasive glucose detection, mainly for wearable devices. The graphene sponge, because of its high surface-to-mass ratio and porous structure, contributes to increasing glucose oxidase binding and electron transportation. Such an arrangement, composed of chitosan and Prussian blue, works at low detecting voltages; it will be capable of real-time glucose monitoring in diabetic patients due to minimal interference because of the other components of the sweat itself.^127^ For laser-induced graphene, various applications of biosensors for health condition monitoring have been made. Laser-induced graphene (LIG), which is prepared by direct laser writing on a polymer, features with a porous structure and good electric and chemical performance, thus it is perfect for sensor applications.^94^ LIG-based sensors have also been used to realize wearables that have been used to detect biomarkers of all types, from ions to proteins and nucleic acids. Graphene-based epidermal sensors, which are ultra-thin and stretchable, have emerged as a new class of devices that adhere conformally to the skin surface for continuous and comfortable physiological signal monitoring. Flexible electronics based on graphene micro-nano electrodes have also been successfully utilized in the design of wearable devices for testing glucose noninvasively in sweat. A noninvasive solution for continuous glucose monitoring is very important for people with diabetes, underlining the potential of wearable sensors in fitness and personalized healthcare.^128^ Graphene-enabled wearable sensors develop the real-time monitoring of health in healthcare by detecting biochemical and biophysical signals. These designs track physiological changes reputedly through mechanical and thermal signals with high validity, providing a noninvasive diagnostic technique that aids continuous health monitoring and personalized healthcare.^129^ Graphene-based wearable biosensors evolved toward noninvasive health monitoring using body fluids such as sweat, saliva, and exhaled breath. Singh *et al.*,^130^ proposed flexibility, conductivity, and biocompatibility of graphene suitable for biomarker detection with regard to a wide range of health conditions. Such graphene-based sensors may enable remote access monitoring of health via the route of body fluids toward preventive healthcare and personalized medicine. Laliberte *et al.*,^131^ developed a flexible GFET on a polyimide substrate and then introduced selectivity through the functionalization of the sensor surface with aptamers specific to IL-6. This wearable biosensor was able to exhibit stable performance even during bending conditions, with the detection range lying in the suitable domain for tracking immune responses in various infections, cancer, and autoimmune diseases. The RF planar resonant structure of the device measures the presence of gases effectively through frequency shift, emphasizing the utility of graphene-based sensors in wearable formats for real-time monitoring of the environment.^132^ A graphene-based nanosensor showed high sensitivity and mechanical durability upon bending and stretching for the purpose of tracking cytokines in biofluids. This sensor is designed as a flexible GFET on an ultrathin polymer substrate for monitoring cytokines such as TNF-*α* and IFN-*γ*, important in diagnostics of inflammation and for tracking chronic diseases.^133^ The wireless integration of graphene-based sensors is being explored to enable seamless data transmission in wearable health systems, allowing real-time updates to smartphones or medical platforms without external connectors. Graphene-based materials are being used in the integrations for energy harvesting in self-powered wearable biosensors, which work without any external power source. This class of self-powered devices generates electricity from biomechanical, biochemical, and solar energies while it actively detects physiological changes for real-time data acquisition in telemedicine and personalized health applications. This points to the role of graphene in the development of sustainable and autonomous health-oriented devices with efficient performance in wearable formats.^134^

### D. Environmental sensors

**FIG. 9.**
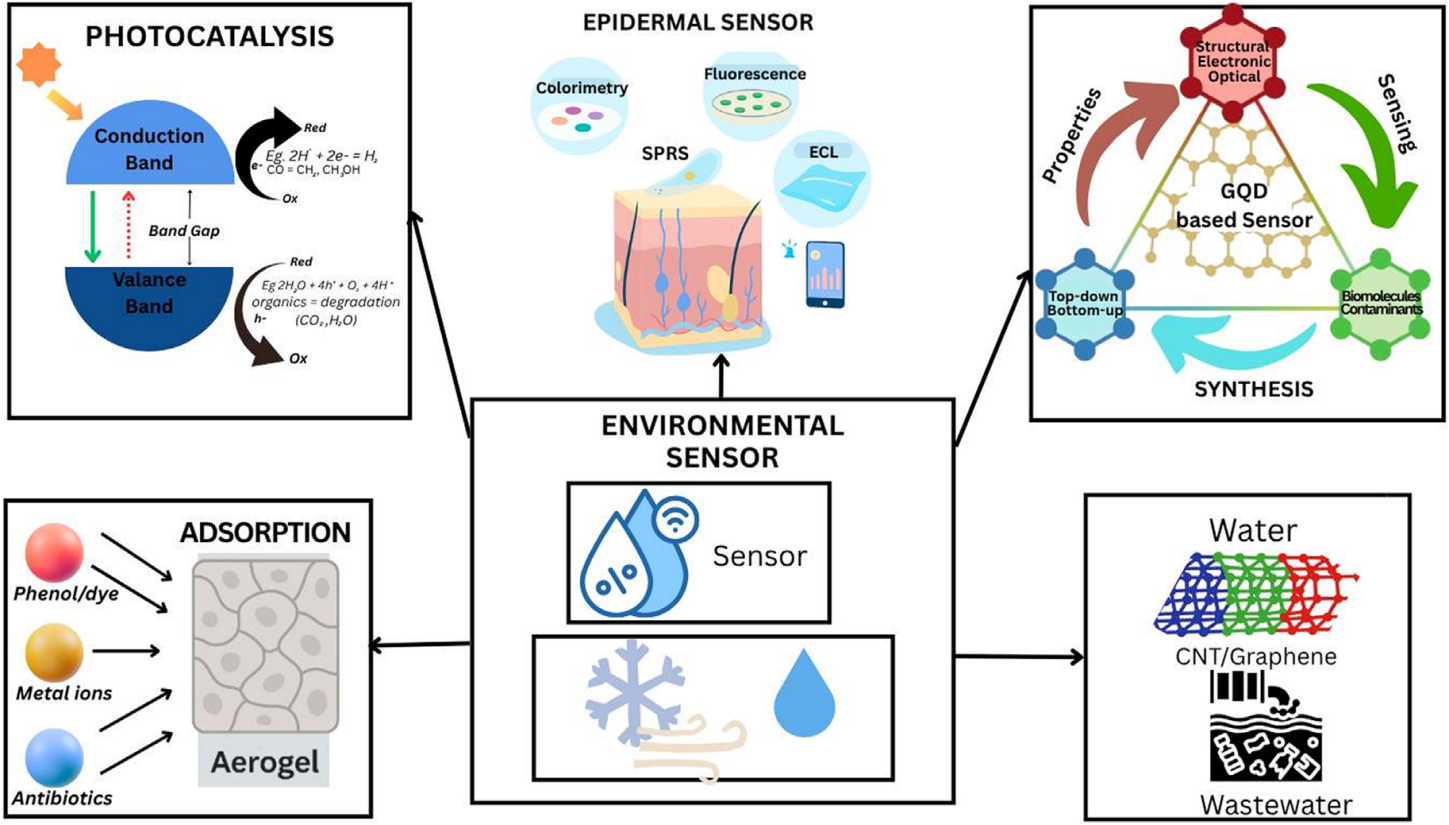
Application of graphene sensors for environmental sensing.

Coupling of TiO_2_ with graphene would enhance the photocatalytic activity of the resulting nanocomposite because of effective charge transport and reduced electron–hole recombination, hence being much more effective in pollutant degradation compared to pure TiO_2_ (refer Table XIII for the environmental remediation studies using graphene and graphene-based composites, covering adsorption, photocatalysis, and pollutant degradation.). Padmanabhan *et al.* discuss design strategies and synthesis techniques to achieve optimal interfacial charge transfer, providing a promising route for environmental remediation.^135^ Wang *et al.* investigated carbon-based nanomaterials such as carbon nanotubes and graphene oxide for environmental remediation, especially in wastewater treatment. These materials are used as adsorbents and catalysts, with unique physicochemical properties applied in the attempt to remove pollutants, thus helping to make environmental management more sustainable. The carbon nanomaterials open up new avenues for the solution of many environmental problems and ecological balance maintenance.^136^ Chaudhuri *et al.* have discussed different 2D/2D composites such as GO/LDHs and GO/MXenes, showing promising performance in adsorption along with antimicrobial activities. Based on structural features and active functional groups, these materials can be further utilized for the removal of metal ions, dyes, and other pollutants in an efficient manner. With a tunable bandgap, GQDs are ideal for detecting hazardous analytes such as heavy metals and pesticides. Chaudhuri *et al.* emphasize GQD-based composites, such as aerogels and nanofibers, which hold the potential for environmental monitoring and pollutant degradation, especially in industrial applications.^137^ Tsang *et al.* presented the role of graphene in green energy, noting its high efficiency in energy storage and conversion, and outlining the future challenges in developing cost-effective, as well as multipollutant remediation methods concerning sustainability.^138^ Facure *et al.* highlighted the veracity of graphene quantum dots in various environmental applications, particularly in sensors, filters, and membranes. The mechanisms and experimental advancements are done for enhancing graphene's performance in antibiotic removal.^139^ Mishra *et al.* discussed the role and advantages of GSF heterojunctions in photocatalysis under visible light irradiation. These heterojunctions possess some unique properties, such as good electron transport and extended light absorption, which make them quite efficient in environmental remediation and energy production.^140^ Li *et al.* did a study on the adsorption and photodegradation of antibiotics from water using different types of graphene and applied them in the treatment of pharmaceutical pollutants present in aquatic systems. Graphene is especially unique in structure for capturing and degrading antibiotic molecules, hence contributing to water purification. It is found light how heteroatom doping and structural modifications can be used in an attempt to improve GSF performance.^141^ Yin *et al.* discussed the chance of CNT/graphene-based composites in organic wastewater treatment. The high specific surface area and tunable properties of these materials make them suitable for adsorbing contaminants even under extreme conditions of wastewater. Mechanistic aspects of adsorption and realistic challenges in scaling up such composites for practical wastewater management have been discussed.^142^ Gao *et al.* highlighted graphene-based aerogels, which are focus entities with tunable properties for pollutant removal. It also gives an overview of their applications in various fields, such as fire protection, drug-delivery systems, and even in the biomedical area. Indeed, the prepared aerogels showed excellent properties related to adsorption capability both for VOCs, NOx, and heavy metals due to their tunable pore size and functional groups. The work exposes the possibility of such aerogels for environmental applications in a scalable way and discusses future challenges toward industrial deployment.^143^ Hossain *et al.* explain the adsorption mechanisms of heavy metals, dyes, and other pollutants onto graphene, besides pointing out several challenges in enhancing the sorption efficiency by composite design, which suggests that continuous improvement of graphene adsorbents is vital for efficient and scalable wastewater treatment.^144^ While conventionally investigated for the degradation of pollutants, graphene-based chemical sensors also find vital applications in biomedical settings with a direct bearing on human health. For instance, photocatalytic activity is improved with the composites of TiO_2_ and graphene toward the decomposition of toxic substances such as atmospheric or aquatic toxins that cause respiratory or endocrine diseases. GQDs and graphene–CNT hybrids exhibit high sensitivity toward the detection of hazardous metals and pesticide residues that cause long-term diseases like cancer and neurodegenerative diseases, as shown in Fig. 9. These graphene-based materials find extensive utilization in the development of wearable or portable biosensor devices for real-time individual exposure analysis. The adsorption capabilities of graphene aerogels and membranes, as explored by Gao *et al.* and Hossain *et al.*, not only contribute to environmental cleanup but also provide the basis for developing implantable or ingestible sensors to monitor and neutralize ingested contaminants. The overlap of environmental remediation materials with health-protective biosensing highlights their significance in preventive biomedical strategies (refer Table XIII for graphene-based biosensor platforms in pharmaceutical and therapeutic monitoring, detailing analytes, sensing strategies, and device performance.).

## IV. CHARACTERIZATION

### A. Raman spectroscopy

Raman spectroscopy has been one of the most sensitive, nondestructive techniques used to characterize unique structural and electronic properties of graphene. It provides information on layer number, defect density, doping level, and edge configuration through the analysis of peak shifts in vibrational modes. This makes it indispensable for both graphene quality control and the development of applications. Raman spectroscopy has proven to be an important technique for investigation into the understanding of graphene properties, both in research and specific industrial applications, each contributing unique insights to such studies. The density of defects in graphene, which is quantitatively measured in many cases through the ID/IG ratio of Raman spectra, greatly impacts its biosensing properties. An increased ID/IG ratio is reflective of greater disorder or defects, but may also function to decrease charge carrier mobility at the same time, potentially increasing sensitivity due to enhanced surface reactivity and adsorption sites, showing the importance of a balance of electrical properties with capacity for functionalization for biosensor design.

Silva *et al.* presented a metrological framework for the determination of layer numbers and stacking order in graphene flakes. In these procedures, both the 2D and G bands under the Raman protocols are used in order to analyze interlayer coupling and layer count. Thus, an automated way using neural-network-based denoising is so important for mass-produced graphene quality control.^145^ Farah *et al.* studied thermally and chemically reduced graphene oxide for structural properties, in particular the ID/IG ratio by Raman spectroscopy, which indicates defect density with respect to different reduction methods.^146^ Li *et al.* demonstrated the crucial usage of Raman spectroscopy concerning layer number, defect density, and stacking order for the characterization of graphene.^147^ Moutinho *et al.* presented Raman spectroscopy for twisted bilayer graphene, including how twisting angles create Moiré patterns, how this affects electronic properties, and how Raman detects specific peaks due to phonons folding within the Moiré superlattice, showing resonance effects at excitation energies dependent on the angle.^148^ Scardaci *et al.* explored the use of Raman to monitor the laser-induced reduction of graphene oxide, examining the D and G band intensity ratios to measure disorder and reduction levels and showed how process parameters, like scan speed and atmospheric conditions, affect graphene oxide's reduction, promoting applications in sensing and optoelectronics.^149^ Jorio *et al.* presented a tutorial on the use of Raman spectroscopy in carbon nanotubes where, in particular, RBM and G-band analyses are performed to assess relevant structural features such as diameter, strain, and defect density. Advanced Raman techniques comprise tip-enhanced Raman scattering (TERS), including high-resolution possibilities for carbon-based materials.^150^ Leng *et al.* developed a technique called Zenith-angle resolved polarized Raman (ZRPR) spectroscopy, which was then used to look into substrate effects on graphene Raman intensities. Properties of the substrate were shown to affect polarization and intensity of the G mode, enriching polarized Raman techniques toward detailed analysis of two-dimensional materials.^151^ Malard *et al.* provided a general overview to distinguish between monolayer and few-layer graphene, characterized by the number of layers, the stacking order, and electronic properties featuring G and G' bands. Raman scattering processes such as first-order and double resonance will elucidate further features of monolayer graphene electronic states, showing how stacking affects phonon interactions.^152^ Wu *et al.* insights the Raman applications for graphene-based materials, highlighting the importance of G and 2D peaks in determining layer numbers, defects, and doping levels. Raman Spectroscopy plays an important role in understanding shear and layer-breathing modes for multilayer graphene and the effects of external factors, such as doping and strain.^153^ Casiraghi *et al.* focused on the Raman characterization of graphene edges, analyzing how zigzag and armchair edge orientations impact D-band intensities and using Raman to probe nanoscale edge structure, a critical factor in graphene-based nanoelectronics.^154^

### B. Optical spectroscopy

Optical spectroscopy is a field of study that deals with the interaction of light and matter. It is thus well-suited for exploring the electronic structure of a material and its energy transitions, including dynamic processes. Recent optical spectroscopies have become particularly important tools for the research of graphene, an extraordinary material both electronically and optically, for investigating its unique behaviors throughout the electromagnetic spectrum, from far-infrared to ultraviolet, bound to further advances in optoelectronic, photonic, and fundamental physical areas.

Branczyk *et al.* presented two-dimensional optical spectroscopy and its applications in an interdisciplinary overview and discussed how 2D electronic spectroscopy (2D ES) records the coherent oscillations in photosynthetic and synthetic materials upon photoexcitation to study the dynamics of energy transfer. It is possible to get information about the nature of inhomogeneous and homogeneous broadening, which becomes a crucial ingredient for microscopic couplings in complex systems and is critically important for understanding energy transfer in graphene-based materials.^155^ Falkovsky explored the optical properties of graphene, namely, its reflectance and transmittance in the optical region. When graphene is doped at low temperatures, the real part of dynamic conductivity saturates as a universal constant, while the imaginary part logarithmically diverges at some transition thresholds. The value of transmittance in the visible range turns out to be practically constant; this fact allows one to draw some conclusions for use in applications where it is necessary to have consistent optical properties for different wavelengths.^156^ Mak *et al.* detailed the graphene's optical spectroscopy, explaining in great detail its electronic structure and extraordinary optical response from the far-infrared to the ultraviolet. The work underlined how graphene's intraband and interband transitions contribute to graphene's optical absorption and how such properties are modified by electrostatic gating. The study also explored plasmonic resonances and excitonic effects in interband absorption and proved that even without a bandgap, graphene has potential applications in the field of optoelectronics.^157^ Riesen *et al.* probed the optical spectroscopy of GQDs, where most of the efforts were concentrated on C132 structures. Riesen *et al.* used absorption, selective fluorescence, and time-resolved spectroscopy and recorded distinct emissions in specific wavelengths, which indicated the existence of different species in the sample. Insight into a near-dark singlet state sheds light on the intrinsic optical properties of GQDs and opens the perspective toward potential applications in optoelectronic devices.^158^ Another experiment, in which graphene was placed side by side with IV-VI semiconductors, has been carried out by Falkovsky, pointing to an unusual behavior of dielectric functions of such systems because of the narrow bandgap and linear electron spectra. This analysis has shown that graphene transmittance in the optical range is constant, being connected with the fine structure constant. Thus, a comparison of graphene with narrow-band semiconductors and underlining its prospects for photonic applications.^159^ Orlita and Potemski studied electronic graphene properties by means of optical spectroscopy, underlining the Dirac-like electronic states in various graphene structures. Th quasi-relativistic behavior is expected in and governed through the Dirac equation for monolayers, bilayers, and multilayers, making graphene an excellent playground for studies of fundamental physics, and very attractive for future electronic applications.^160^ Schoche *et al.* studied the optical properties of GO and rGO using spectroscopic ellipsometry. Dynamically measured during reduction, the changes in optical constants and thickness allowed them to show that the anisotropic properties of GO become isotropic in rGO. These results showed exactly how functional groups of oxygen take part in the variation of the optical properties and helped do the precise characterization necessary for device fabrication.^161^ Weber *et al.* used spectroscopic ellipsometry to determine the optical constants and thickness of monolayer graphene on silicon. With B-splines for parameterization, correct measurements of thickness and optical constants over a wavelength range proved ellipsometry's precision for probably reliable graphene characterization in industrial applications.^162^ Mak *et al.* further investigated FLG by infrared conductivity measurements in which distinct spectral features and systematic changes in energy scaling with thickness were identified. These results are in good agreement with the zone-folding model and prove that in FLG the electronic states stem from bulk graphite, with boundary-imposed massless and massive fermion components, illustrating the tunable electronic properties of FLG for optoelectronic applications.^163^ Hochstrasser *et al.* extended the applicability of optical spectroscopy by employing ultrafast laser pulses in two-dimensional spectroscopy, allowing real-time observation of molecular motions in complex systems. Based on this principle, this technique borrows from NMR spectroscopy, providing great resolution for studies that might be valuable in dynamics associated with structural changes in graphene and other molecular assemblies.^164^

### C. Scanning electron microscopy (SEM)

Scanning electron microscopy nowadays has played an important role in graphene studies by offering imagery of high resolution with deep analysis of nanostructures and growth mechanisms. By utilizing the way electrons interact with material surfaces, SEM thus allows for imaging of graphene surface topography, making it form into specific growth patterns or interact with other elements or substrates. The SEM is complemented with other techniques, like EDS or even better, super-resolution microscopy, which greatly extends its utility for such a technique in understanding its composition, structural details, and respective applications of graphene in several scientific fields.

Mohammed *et al.* conducted an experiment on SEM, its constituent elements, the working principles involved, and magnification capabilities up to 300 000×, thereby making the SEM an instrument of excellence in the study and investigation of graphene nanostructures. Coupling SEM with energy-dispersive x-ray Spectroscopy secures qualitative and quantitative analysis of compositional data and enables precise characterization of graphene surface, structure, and composition as demanded by materials science applications.^165^ Zhou *et al.* highlighted the principles of SEM, with a particular focus on high-resolution versions for microstructural morphological studies, such as those of graphene. These authors emphasized that in SEM, a very fine probe of high-energy electrons interacts with the surface of a specimen to generate a signal, which thus results in the generation of finely detailed images. In this respect, it is especially fitted for providing nanoscale imaging of graphene.^166^ Leamy noted the application of SEM during the study of semiconducting materials by the methods of Electron Beam-Induced Conductivity (EBIC). Such a method enabled them to detect inhomogeneities in electrical properties and provided important insights for an analysis of such complex materials as graphene. EBIC imaging supplies information about carrier lifetimes, diffusion lengths, and defect energy levels, which is useful information in understanding graphene electronic applications.^167^ *In situ* SEM has been used by Wang *et al.* to observe graphene growth on copper substrates by CVD. The authors record the entire process of CVD, visualizing nucleation, growth, and cooling of graphene, showing how copper grain orientation influences surface dynamics. The findings especially stress the capability of SEM to monitor real-time growth mechanisms, which is an essential prerequisite for understanding substrate interactions and further optimization of graphene synthesis.^168^ Sohn *et al.* analyzed two-materials composed of graphene through aberration-corrected STEM. The work emphasized AC-STEM sub-angstrom precision of imaging that is enabled by viewing atomic structures and defects in graphene. It also underlined the fact that the low voltage imaging is so essential in avoiding electron beam-induced damage, important in materials as sensitive as graphene.^169^ Zan *et al.* used atomic resolution STigate metal–graphene interactions, finding that metals like gold and iron form nanoclusters on few-layer graphene. Chromium atoms, however, bonded more strongly and facilitated the dissociation of carbon–carbon bonds. The findings provide insights into how metal type and graphene layer structure affect interactions, with implications for electronic applications.^170^ Takahashi *et al.* conducted an *in situ* SEM study on the growth of polycrystalline nickel, observing that monolayer graphene formation is dependent on the nickel grain orientation. The real-time imaging by SEM allowed them to investigate growth features that were specific to nickel substrates, which are important for controlled graphene synthesis.^171^ Stoll and Kolmakov also discussed the use of graphene wafers for environmental SEM in liquid environments. The work allowed SEM imaging with high resolution of specimens, such as colloidal nanoparticles in water, that showed artifacts related to beam-induced radiolysis and membrane stability issues. This technique has extended the capability of SEM to liquid environments—an area important for the imaging of graphene in more challenging situations.^172^ Dyck *et al.* utilized scanning transmission electron microscopy (STEM) to move atoms within a grain by using an electron beam to inject silicon atoms into the sample selectively and produce controlled defects. This pioneering atom-by-atom assembly showcases STEM's potential for precise nanofabrication and chemical manipulation at the atomic scale within graphene structures.^173^ Wojcik *et al.* explored graphene-enabled SEM combined with super-resolution microscopy and a graphene membrane to preserve hydrated biological cells under high vacuum. This method allowed detailed imaging of cellular structures, including actin filaments, in their natural state, opening new possibilities for biological imaging at the nanoscale and demonstrating graphene's versatility in extending SEM's scope.^174^ SEM is capable of providing high-resolution imaging of graphene morphology and composition analysis when combined with methods such as EDS or STEM. SEM is limited in its applicability toward the development of biosensors, particularly for soft-matter and biosystems, due to certain restrictions. One such restriction is the need for high-vacuum conditions that inhibit the analysis of hydrated samples and soft biosystems commonly involved in biosensor testing. Moreover, the high-energy electron beam of SEM is prone to cause damage in the sample of interest, such as fragile nanostructures or thin graphene films, and might change the properties of the material while being imaged. Such restrictions become more significant in the case of implantable and wearable biosensor characterization, as stability against the environment and integrity of native biosystems play critical roles. SEM is not capable of providing direct insight into the properties, such as stiffness and adhesion, of the material, which is commonly required in the analysis of sensor-tissue biointerfacing in bioengineering. Therefore, SEM is superior for morphological and elemental analysis but is restricted by its approach toward biosensor characterization in terms of biosocompatibility and in situ observation.

### D. Transmission electron microscopy (TEM)

Transmission electron microscopy is an important technique to view graphene in atomic and nanoscale detail, thus providing enormous detail regarding its defects, chemical composition, and structural behavior under changing conditions. The high-resolution capability of TEM enables electronic, mechanical, and chemical property analysis of graphene at the atomic level. This allows an extension of graphene applications in electronics, energy storage, and materials science. The following studies illustrate the versatility and precision of TEM when it comes to analyzing many of the unique features and behaviors of graphene.

Robertson *et al.* carried out research on atomic-resolution imaging of graphene by aberration-corrected TEM. The authors have demonstrated that AC-TEM can visualize atomic-scale defects, grain boundaries, and impurity dopants—all very relevant to electronic and mechanical properties of graphene. Vacancy defects and bond rotations observed have shown a way in which AC-TEM can assist in tailoring the atomic configuration to optimize graphene for electronic applications.^175^ The High-Resolution Transmission Electron Microscopy (HRTEM) analysis of Shalaby *et al.* was devoted to the lattice structure of RGO and demonstrated the presence of clear interplanar distances and minor impurities of graphite in RGO. The authors paid attention to structural differences from pristine graphite using selected area electron diffraction (SAED) and underlined unique features of this material, which is important for materials science and different technological applications.^176^ Pantelic *et al.* explored the use of graphene as a sample support in TEM graphene's atomic thinness, stability, and conductivity, which enhance image contrast and minimize background noise in TEM imaging, and illustrated graphene's benefits as an ideal support material for both cryo-electron microscopy in biological research and nanoscale visualization of defects in materials science.^177^ Textor and de Jonge developed graphene liquid cells for TEM; these cells encapsulate liquid samples between graphene sheets. The method preserves liquid-phase phenomena while enabling high-resolution imaging. The applications of the work ran from biological and material studies, as this addressed how to observe nanoscale interaction in liquid environments, something that is not easily done with standard TEM conditions.^178^ Politano *et al.* used TEM combined with electron energy loss spectroscopy (EELS) to investigate plasmonic modes in graphene. By examining *π* and *π* + s plasmons at atomic resolution, it is found that graphene's plasmonic properties vary with layer thickness, advancing its optical and electronic applications. It demonstrated TEM-EELS's utility in analyzing the plasmonic behavior of 2D materials at the nanoscale.^179^ Zan *et al.* investigated metal interactions with suspended graphene using high-resolution TEM, noting that metals like gold and chromium cluster on hydrocarbon-contaminated graphene. Some metals, such as aluminum and chromium, facilitated graphene etching under specific conditions, enabling controlled surface modifications. TEM can elucidate metal—graphene interactions, providing insights into potential electronic applications.^180^ Liu *et al.* explored lithiation and delithiation and their mechanical deformation of graphene nanoribbons. Lithiation resulted in the formation of a lithium oxide layer over the surface of GNR, and the lithiated GNRs were mechanically robust as compared to multi-walled carbon nanotubes, which are brittle in nature. This work highlights the possibility that graphene can be used as a durable anode material in the case of lithium-ion batteries.^181^ Lu *et al.* followed up with *in situ* TEM studies of graphene nanoconstrictions fabricated using electron beam nanosculpting. It is observed GNC structural changes under current annealing within TEM and correlated these changes with electronic properties, identifying high conductance in nanoscale graphene nanoconstrictions (GNCs). This work underscores TEM's ability to correlate structural and electronic properties, which is beneficial for nanoelectronics.^182^ Lehtinen *et al.* demonstrated TEM's use in visualizing vacancy defects in graphene by encapsulating it within protective graphene layers, thereby reducing beam-induced damage. This approach enabled clear imaging of monovacancies, with implications for studying defect-mediated properties relevant to spintronics. The method illustrates how TEM can be adapted to reduce contamination and enhance the visualization of intrinsic defects.^183^ Chen *et al.* provided an overview of *in situ* TEM techniques for 2D materials, describing real-time observation of structural evolution under external stimuli. The work highlights recent advancements in achieving high spatial and temporal resolution, crucial for understanding graphene's behavior in nanoscale devices.^184^

### E. Atomic force microscopy (AFM)

Atomic force microscopy represents one of the most important tools for investigating graphene due to its high-resolution surface imaging and measurement capabilities. There is a sharp tip moving over the sample surface in AFM, sensing the interaction between the probe and the sample for generating nanoscale images. Such techniques allow one to precisely analyze the morphology of the graphene surface, thickness, mechanical properties, and frictional behavior. Because of this, AFM becomes very important in undertaking an optimization of its use for applications related to electronics, nanotechnology, and materials science.

Jiang and Zhu measured adhesion energy between graphene and various substrates by using a microsphere tip for AFM and the Maugis–Dugdale model. According to the study, the adhesion on SiO_2_ is 0.46 J/m^2^ and on copper, 0.75 J/m^2^, only due to van der Waals attraction. This technique has been promising in analyzing the interfacial properties of graphene in handling applications for device fabrication.^185^ Eigler *et al.* combined AFM with scanning Raman microscopy to assess the morphology and defect density in graphene derived from GO. AFM measured height profiles of monolayer and bilayer regions, while Raman spectroscopy correlated defect density to structural quality. This integration allowed detailed visualization of graphene flakes, highlighting AFM's role in assessing GO quality and providing a basis for comparing treated graphene samples.^186^ For example, Lindvall *et al.* reported the mechanical cleaning of graphene using AFM in contact mode. The contaminants were mechanically removed by scanning an AFM tip in a broom-like fashion, which resulted in an atomically smooth surface with a root mean square roughness of 0.12 nm. This method significantly enhanced the graphene's electronic properties, proving AFM's effectiveness in improving sample quality for electronic applications.^187^ Rasuli *et al.* examined the mechanical properties of few-layer graphene cantilevers (FLGC) using AFM and density functional theory (DFT). The AFM force-displacement curves measured Young's modulus at around 0.7 TPa, with variations observed at zigzag vs armchair edges. AFM's ability to assess mechanical robustness, demonstrating graphene's suitability for nanoelectromechanical systems, is highlighted.^188^ Lavini *et al.* used AFM phase imaging to characterize epitaxial graphene films on silicon carbide. The study found that phase shifts correlated with graphene layer thickness, distinguishing monolayer, bilayer, and buffer layers. AFM covers specifics of changes in surface properties and energy dissipation, thereby helping to characterize variations in nanoscale graphene films without the need for further complex imaging techniques.^189^ Lin *et al.* investigated the friction and wear characteristics of multi-layer graphene films using AFM. The results indicated that graphene's friction on silicon substrates was substantially lower than that of bare silicon. Wear tests showed moderate resistance to wear after repeated cycles, highlighting graphene's low-friction properties, which are beneficial for applications requiring durability under sliding contact.^190^ Filleter and Bennewitz studied frictional properties in single- and bi-layer graphene on SiC(0001) by AFM. Friction was found to be nearly twice as large on single-layer layer compared to bi-layer graphene, coupled to the discussed electron–phonon coupling. A transition from atomic stick-slip friction to ultralow friction was observed for low loads. This research demonstrates AFM's capacity to differentiate frictional properties based on layer thickness, relevant for tribological applications.^191^ Weng *et al.* applied AFM for local oxidation nanopatterning of graphene, creating insulating trenches and nanodevices with high precision. By adjusting lithography conditions, we could fabricate nanoscale features, such as a 25-nm-wide nanoribbon. This AFM-based technique, which has been developed below, presents a universal method for nanofabrication and is highly important for electronic applications requiring controlled structural modifications.^192^ Giesbers *et al.* fabricated isolating trenches by local anodic oxidation using AFM. Indeed, for nominal widths as low as 30 nm, the study proved the capability of AFM to structure graphene with precision suitable for nanoscale device fabrication, such as quantum dots and point contacts. This highlights AFM's potential in creating and manipulating nanoscale features.^193^ Yao *et al.* developed a histogram-based AFM method to measure graphene thickness accurately, minimizing contamination effects. By optimizing parameters like amplitude setpoint, the study enhanced thickness measurement reliability, crucial for applications where graphene's layer count affects electronic and thermal properties.^194^ Inspite of its high-resolution surface images and nanoscale surface-characterization capabilities, AFM is fundamentally limited in its inability to probe beneath surface-level defects. Since AFM simply evaluates the surface of a sample using mechanical contact of a cantilever tip with the sample surface, it is limited to the collection of surface topography and is not capable of sensing anything beneath the surface layer. This is of particular importance in biosensor fabrication when impurities within the substrate below the surface level can critically influence the sensor's electrical stability and reliability, and its compatibility with biology. In the case of implant biosensors, for instance, unseen inconsistencies in the structure within buried interfaces have the potential to cause signal drift, material deterioration, and poor tissue-to-device integration, with the consequences of all compromising long-term performance. Thus, as critical as AFM is to morphological and mechanical evaluations, its inability to probe below the surface is a fundamental limitation when ascertaining the integrity of the graphene biosensor structure.

## V. LIMITATIONS AND FUNCTIONAL ROLE OF GRAPHENE

The distinctive characteristics of graphene, such as its atomic thickness, elevated carrier mobility, superior electrical conductivity, and extensive specific surface area, render it a compelling material for signal transduction in biosensors.^195^ It is crucial to emphasize that graphene, on its own, lacks intrinsic selectivity or biological recognition capabilities. In biosensor systems, the bioreceptor—comprising an enzyme, antibody, aptamer, or peptide—serves as the principal factor influencing specificity and affinity for the target analyte. Consequently, graphene's function should be acknowledged as auxiliary and signal-amplifying rather than fundamental in analyte detection.^196^ In electrical biosensors such as graphene field-effect transistors (GFETs), graphene serves as a highly sensitive medium for sensing alterations in surface potential during biomolecular interactions. The selectivity of such a device for a virus, protein, or nucleic acid is solely determined by the surface-bound functional molecule (e.g., anti-spike antibodies for SARS-CoV-2).^197^ In the absence of this functionalization, graphene functions as a versatile conductor or semimetal, failing to differentiate between molecular species. Assertions suggesting that “graphene is sensitive to viruses” are factually inaccurate and could result in misinterpretation unless explicitly contextualized regarding surface changes. Furthermore, the fabrication and biofunctionalization processes substantially affect the performance and reproducibility of graphene-based biosensors. The elevated surface reactivity of graphene, although advantageous for the attachment of sensing moieties, may potentially result in nonspecific adsorption or degradation in ambient settings. The variability in graphene quality, arising from production techniques like chemical vapor deposition (CVD), liquid-phase exfoliation, or electrochemical exfoliation, can lead to variations in sensor performance. These variances may present as alterations in defect density, doping concentration, or layer count, all of which can profoundly influence electrical response and sensitivity.

A significant restriction pertains to the difficulty of achieving scaled and uniform biofunctionalization. Immobilizing bioreceptors on graphene surfaces frequently entails intricate chemistries (e.g., EDC/NHS coupling, *π*–*π* stacking, or covalent grafting), and preserving receptor orientation and functionality after attachment can be challenging. These difficulties can affect both the signal-to-noise ratio and long-term stability. Furthermore, the efficacy of biosensors may diminish with time as a result of environmental influences such as humidity, pH, and oxidation, which can modify the graphene surface or denature the affixed bioreceptors. Notwithstanding these constraints, graphene consistently provides notable benefits in improving signal transduction, lowering detection thresholds, and facilitating the downsizing of sensing apparatuses. Its suitability with microfabrication and integration into flexible and wearable electronics presents novel prospects for point-of-care diagnostics and real-time health monitoring. Ultimately, it is the interplay between graphene and the functional bio-recognition architecture—not graphene in isolation—that dictates the efficacy of a biosensing platform. Consequently, in the realm of biosensor creation, graphene ought to be viewed not as the origin of specificity, but as a high-performance signal transducer that enhances and amplifies the efficacy of meticulously selected biorecognition elements and detection methodologies.

The clinical application of graphene-based biosensors necessitates both rigorous scientific validation and compliance with strict regulatory norms. Notwithstanding encouraging outcomes in *in vitro* and preclinical investigations, few graphene-based devices have advanced to clinical use due to obstacles including biocompatibility, long-term safety, scalability of production, and consistency of sensor efficacy. Regulatory agencies, including the U.S. Food and Drug Administration (FDA) and the European Medicines Agency (EMA), need thorough reviews, encompassing toxicological assessments, human clinical trials, and adherence to Good Manufacturing Practice (GMP). Furthermore, graphene materials must satisfy classification standards as medical devices or combination items, contingent upon their intended application. To promote clinical acceptance, developers must consider integration with current diagnostic workflows and demonstrate cost-effectiveness. Implementing standardized techniques for graphene functionalization, biosensor calibration, and performance evaluation will be essential for securing regulatory approval and facilitating the widespread clinical application.

## VI. FUTURE DIRECTIONS AND CONCLUSION

Graphene-based biosensors have emerged as a very promising platform in sensing technology, owing to their distinctive amalgamation of physical, electrical, and chemical capabilities. This review emphasizes how graphene improves the performance of electrical, electrochemical, and optical biosensors due to its exceptional electron mobility, extensive surface area for biomolecule immobilization, and mechanical flexibility, making it suitable for integration with wearable and portable devices. Graphene's capacity for ultra-sensitive signal transduction facilitates the detection of biomarkers at minimal concentrations, essential for early disease diagnosis, environmental pollution monitoring, and food safety verification. We have examined various fabrication techniques—namely, CVD, liquid-phase exfoliation, and electrochemical exfoliation—that are scalable and appropriate for industrial production. Furthermore, we elucidated that characterization techniques, such as Raman spectroscopy, AFM, SEM/TEM, and XPS, are crucial for verifying the quality of graphene before sensor integration. Notwithstanding its benefits, considerable obstacles persist. Graphene lacks inherent selectivity, and the attainment of particular detection relies only on surface functionalization with biological recognition components, including antibodies or aptamers. Furthermore, heterogeneity across batches in graphene manufacturing may result in uneven sensor outputs. Environmental instability, particularly in non-encapsulated devices, along with performance deterioration over time, impedes economic viability. Moreover, defined techniques for functionalization, packaging, and calibration remain absent throughout the area.

Anticipating the future, three critical trajectories are set to influence the development of graphene-based biosensors:

• Hybrid sensing platforms: The integration of graphene with an additional two-dimensional material may provide synergistic electrical or optical characteristics, facilitating multimodal and multiplexed biosensing.
• Graphene's mechanical toughness and biocompatibility render it ideal for skin-wearable and implanted biosensors. Future research should concentrate on the integration of energy-harvesting devices with wireless data transmission to develop completely autonomous platforms.
• Incorporating graphene biosensors into Internet of Things (IoT) networks and utilizing artificial intelligence for signal interpretation will facilitate intelligent, real-time, large-scale diagnostics and environmental monitoring, applicable in customized medicine and precision agriculture.
• Commercial translation necessitates consistent large-area graphene manufacturing, dependable device encapsulation, long-term storage stability, and regulatory validation to connect laboratory performance with real-world applications.

Although graphene-based biosensors are still developing, their proven sensitivity, versatility, and integration potential position them at the vanguard of next-generation biosensing technologies. Ongoing advancements in materials processing, device engineering, and system-level integration will be essential to effectively harness their influence on healthcare, environmental protection, and industrial safety.

## Data Availability

The data that support the findings of this study are available from the corresponding author upon reasonable request.

## APPENDIX: Supplementary Tables

Table IV: Summary of key studies on mechanical exfoliation of graphene-based materials, including author names, publication years, and main findings. Table V: Summary of selected works on liquid-phase exfoliation methods for producing graphene and other 2D nanosheets, with details on mechanisms and performance. Table VI: Overview of major advances in chemical vapor deposition (CVD) of graphene, highlighting growth techniques, process parameters, and resulting film properties. Table VII: Comparative summary of research on electrochemical exfoliation of graphite into graphene, including electrolytes, voltages, and functionalization outcomes. Table VIII: Key publications on microwave-assisted exfoliation of graphite for graphene synthesis, detailing solvents, power settings, and product quality. Table IX: Applications of graphene-based materials in cancer diagnostics, summarizing biosensor designs, targets, and analytical performance metrics. Table X: Graphene-based biosensors and nanosensors for food safety and contaminant detection, summarizing analytes, transduction principles, and sensitivities. Table XI: Graphene-based biosensor platforms in pharmaceutical and therapeutic monitoring, detailing analytes, sensing strategies, and device performance. Table XII: Development of wearable graphene devices for biochemical and physiological monitoring, including sensor configurations and detection limits. Table XIII: Environmental remediation studies using graphene and graphene-based composites, covering adsorption, photocatalysis, and pollutant degradation.

**TABLE IV.** Summary of key studies on mechanical exfoliation of graphene-based materials, including author names, publication years, and main findings.

Author Name	Paper Title	Publication Year	Findings/Results
Baqiya *et al.*^16^	Structural study on graphene-based particles prepared from old coconut shell by acid–assisted mechanical exfoliation	2020	In this study, graphene-based particles synthesized from burnt coconut shell through the process of mechanical exfoliation are discussed. With the assistance of HCl-assisted processing, particle sizes in the range of 1.42–4.99 nm were recorded as evidenced by SAXS, TEM, and AFM characterizations. XRD, Raman, etc., characterization showed that reduced graphene oxide with improved size reduction had been produced and highlighted a simple, biomass-based approach to the attainment of nanoscale graphene materials.
Chen *et al.*^25^	Effects of hybrid surfactants on the quality and yield of graphene using a novel electrochemical-mechanical exfoliation process	2022	This work developed an EME process using hybrid surfactants for the preparation of high-quality GNSs. A yield of 19.68% was realized, while the average diameter and thickness of GNSs were determined as 5.2 *μ*m and 2.4 nm, respectively. Characterization with various techniques indicated that the prepared graphene nanosheets have low defects, high crystallinity, and a reduced ratio of oxygen-to-carbon atoms.
Chow *et al.*^28^	Mechanical Exfoliation of Expanded Graphite to Graphene-Based Materials and Modification with Palladium Nanoparticles for Hydrogen Storage	2023	The presented study has optimized the exfoliation of EG into graphene-based materials, ShEG and sEG, for hydrogen storage. Functionalization with Pd-NP significantly enhances the hydrogen uptake-release process and allows reaching as high a value as 31.05 mC cm^−2^ for Pd-sEG. This kind of study would point out that, in such a way, a proper relationship exists between nanoparticle size (or surface area) and hydrogen capacity, indicating a cost-effective way to enhance solid-state hydrogen storage performance.
Kurniawan *et al.*^29^	Mechanical Exfoliation of Reduced Graphene Oxide From Old Coconut Shell as Radar Absorber in X-Band	2019	The temperature of heating and chemical exfoliation was the variable used to study reflection loss of the synthesized r-GO derived from coconut shell. The samples thermally treated at 400 °C using a 1:10 H_2_SO_4_ solution yielded the highest reflection loss of −7.186 dB at 10.48 GHz with electrical conductivity of 1.075 × 10^−3^ S/cm, hence confirming that r-GO was formed and its performance was enhanced in the process.
Myroniuk *et al.*^22^	Mechanical exfoliation of graphite to graphene in polyvinylpyrrolidone aqueous solution	2023	The work details the achievements in low-cost and industrially scalable graphene production via shear-assisted liquid-phase exfoliation of graphite in organic solvents using a kitchen blender. The produced graphene material is of high quality and low defect density and requires proper solvent selection to be sufficiently stabilized.

**TABLE V.** Summary of selected works on liquid-phase exfoliation methods for producing graphene and other 2D nanosheets, with details on mechanisms and performance.

Author Name	Paper Title	Publication Year	Findings/Results
Li *et al.*^30^	Mechanisms of liquid-phase exfoliation for the production of graphene	2020	The three stages of ultrasonic liquid-phase exfoliation of graphite into graphene are discussed in this study: (i) flake rupture and kink band striation, (ii) crack and intercalation of solvent, which results in unzipping of the graphite strips, and finally (iii) graphene exfoliation. These will also provide insights into optimizing graphene's lateral dimensions and thickness vs yield for large-format applications.
Liu and Li^43^	Study on ultrasound-assisted liquid-phase exfoliation for preparing graphene-like molybdenum disulfide nanosheets	2020	This work optimized the ultrasound liquid exfoliation of graphene-like molybdenum disulfide using 1-dodecanethiol and chloroform. Under the optimal condition, with a 1:1 solvent ratio and 12 h of processing, high-concentration and few-layer nanosheets of graphene-like molybdenum disulfide were obtained. The resulting nanosheets can be dispersed in different organic solvents by the solvent exchange method. The preparation methods of these nanosheets were compared based on their performances and scalability, and their applications were discussed accordingly.
Ma and Shen^36^	Exfoliation of graphene nanosheets in aqueous media	2020	This review discusses recent advances in environmentally friendly aqueous exfoliation approaches for graphene nanosheets. Mainly, liquid-phase and electrochemical exfoliation methods are emphasized as sustainable and scalable methods. The use of water as the dispersion medium circumvents the use of toxic types of solvents that allow effective exfoliation and stabilization in aqueous systems cognizable for cost-effective and eco-friendly mass production of high-quality graphene.
Paolucci *et al.*^40^	Sustainable liquid-phase exfoliation of layered materials with nontoxic polarclean solvent	2020	The present work describes sonication-assisted liquid-phase exfoliation of graphene and other 2D materials by employing Rhodiasolv Polarclean, a green and biodegradable solvent. Accordingly, compared to NMP, Polarclean increased the yield of few-layer graphene flakes with reduced defects and large lateral size by ∼350%, up to ∼10 *μ*m with I(D)/I(G) = 0.07. Its eco-friendly nature enables sustainable, large-scale production toward diversified applications.
Alzakia and Tan^42^	Liquid-exfoliated 2D materials for optoelectronic applications	2021	This review presents the recent progress in liquid exfoliation for preparing 2D nanosheets toward optoelectronic applications and focuses on MSM photodetectors. Liquid-phase exfoliation represents a low-cost, scalable technique toward flexible and wearable devices. Integration with materials such as quantum dots and nanowires greatly improves the performance of such optoelectronic devices. The light detection strategy is discussed to improve the overall efficiency in photodetectors based on 2D nanosheets.

**TABLE VI.** Overview of major advances in chemical vapor deposition (CVD) of graphene, highlighting growth techniques, process parameters, and resulting film properties.

Author Name	Paper Title	Publication Year	Findings/Results
Jia *et al.*^51^	Superclean growth of graphene using a cold-wall chemical vapor deposition approach	2020	This work demonstrates that gas-phase reactions are suppressed in the cold-wall CVD system, hence allowing superclean and controllable graphene film growth. Consequently, graphene films have superior optical and electrical properties for transparent electrodes and substrates of epitaxial growth. This technique offers a promising path toward the industrial-scale production of high-quality graphene films and solving contamination-related problems.
Mishra *et al.*^58^	Experimental advances in charge and spin transport in chemical vapor deposited graphene	2021	This review focuses on the major developments achieved within wafer-scale CVD graphene synthesis concerning large-grain growth and charge/spin transport improvement. The great successes in CVD graphene include high carrier mobility, ballistic transport at the micrometer scale, and room temperature robust spin diffusion over tens of micrometers. These make CVD graphene ultra-promising for next-generation nanoelectronics and spintronics while pointing out scalability and performance enhancements.
Nandanapalli *et al.*^50^	User-friendly methodology for chemical vapor deposition–grown graphene-layers transfer: Design and implementation	2021	Herein, a green wet-chemical transfer technique is presented to transfer the CVD-grown graphene monolayer onto Si/SiO_2_ substrates, ensuring high chemical purity and homogeneity with excellent electrical properties. Graphene/insulator/metal configuration devices showed high electrical conductivity with low leakage current. The scalability of the proposed technique without sustaining chemical damage in the transferring process and without any fabrication failure allows easy extension to other 2D materials.
Su and Li^53^	Effect of plasma-enhanced chemical vapor deposition (PECVD) graphene content on the properties of EPDM/graphene composites	2021	This work demonstrates that the addition of 0.5% of plasma-enhanced CVD-fabricated graphene platelets increases the dielectric constant of EPDM-based elastomers by 230% (from 2.0 to 6.6 at 10^3^ Hz), while electric resistivity is barely affected. Besides, the complex viscosity decreased 1.9 times and the dielectric constant-to-Young's modulus ratio increased 180%, which means that dielectric performance was enhanced without losing processability.
Wang *et al.*^46^	A review of graphene synthesis at low temperatures by CVD method	2021	The present review focuses on recent progress made in the synthesis of high-quality graphene via CVD at low temperature (<600 °C) by overcoming conventional, costly and unscalable methods. It further compares critically the quality of graphene, the number of layers, and the domain size for different precursors, substrates, and CVD techniques, including plasma- and catalyst-enhanced ones. Homogeneity of quality control, large-scale production, and increasing substrate compatibility will remain challenges for the future.

**TABLE VII.** Comparative summary of research on electrochemical exfoliation of graphite into graphene, including electrolytes, voltages, and functionalization outcomes.

Author Name	Paper Title	Publication Year	Findings/Results
Aghamohammadi *et al.*^62^	Recent advances in one-pot functionalization of graphene using electrochemical exfoliation of graphite: A review study	2020	The review summarizes recent progress on large-scale, one-step electrochemical exfoliation toward functionalized graphene and graphene/nanoparticle composites. The approaches most employed pertain to electrode modification, often from pre-coiling with wires of metals, and sometimes electrolyte optimization by adding FeCl_3_ and Na_2_S_2_O_3_. While much of the major challenges pertaining to high functionalization degree scaling remain, efforts at optimizing setup design, electrode preparation, and the use of the best electrolyte mixture will lead to an industrially applicable process.
Ali *et al.*^60^	Synthesis and characterization of high-quality multi layered graphene by electrochemical exfoliation of graphite	2022	This study optimizes several parameters of the electrochemical exfoliation in the production of high-quality multilayer graphene, including the type of electrolyte used together with its concentration and DC voltage. The synthesized graphene-using 0.3M H_2_SO_4_ and 30% KOH at +10 V DC-exhibits a wrinkled, few-layer morphology with attached functional groups (C-O, C-OH) and excellent structural characteristics.
Bilgiç^63^	Reduced Graphene Synthesis via Eco-Friendly Electrochemical Exfoliation Method	2024	This work is related to a greener and cost-effective route in the production of graphene involving electrochemical exfoliation using whey as an electrolyte. The process yielded stable graphene dispersions without surfactants or functionalization, taking advantage of stored acidic wastewater at +4 °C to further promote exfoliation. XRD confirmed the successful synthesis of graphene, hence a more viable and sustainable alternative toward the production of graphene and graphene oxide.
Lee *et al.*^69^	Role of anions on electrochemical exfoliation of graphite into graphene in aqueous acids	2020	This work recognizes sulfate-containing electrolytes as the most efficient for anodic electrochemical exfoliation of graphite owing to its reversible intercalation behavior together with high repulsive binding energy between graphene sheets, which allows the diffusion and oxidation of water molecules, increasing the exfoliation efficiency. The insights derived from this work detail the significant influence of anion-graphite interactions in optimizing electrochemical graphene production.
Loudiki *et al.*^65^	Preparation of graphene samples via electrochemical exfoliation of pencil electrode: Physico-electrochemical Characterization	2022	This work reported an easy method of electrochemical exfoliation at ambient temperature for synthesizing a stable GO/rGO mixture using pencil graphite. It achieved uniform dispersion in 35 min of sonication. Such characterization confirmed that GO and rGO coexisted, each having distinct d-spacings. Electrochemical analysis showed controllable oxidation levels, hence rendering such material promising for sensing applications because of its enhanced redox performance and stability.

**TABLE VIII.** Key publications on microwave-assisted exfoliation of graphite for graphene synthesis, detailing solvents, power settings, and product quality.

Author Name	Paper Title	Publication Year	Findings/Results
Gürünlü *et al.*^78^	One Pot Synthesis of Graphene through Microwave Assisted Liquid Exfoliation of Graphite in Different Solvents	2022	The study presented a prompt microwave-assisted exfoliation method to produce graphene using various solvents. XRD confirmed the number of layers was between 2 and 25, with up to two layers obtained for G-DMSO, G-EG, and G-OCTA. For G-DMF, the conductivity reached as high as 22 S/m, consistent with expectations from the dipole moment of the solvents that fell within a window of 2–4 D. FTIR and UV-Vis spectra were similar to commercial graphene, confirming good quality synthesis.
Jakhar *et al.*^77^	Microwave reduction of graphene oxide	2022	This review covers a cost-effective, chemical-free method to produce graphene from GO through microwave reduction. It describes solid-state microwave reduction and solvent-assisted microwave reduction, emphasizing accelerated volumetric heating. Hybrid microwave heating enhances the efficiency of reduction. In this way, this method can overcome the limitations of chemical and thermal methods to provide a scale-up, eco-friendly route to graphene-based material production.
Ji *et al.*^85^	Scalable fabrication of holey graphene nanosheets by electrochemical intercalation and microwave-assisted expansion of graphite	2021	HGNSs have been developed by using dual-electrochemical intercalation and microwave-assisted expansion of graphite. Obtained HGNSs contain about 5–10 layers with ultralow defects (ID/IG < 0.07), possessing high conductivity of 691 S/cm. The hierarchical pores in HGNS, micropores (∼1.13 nm), mesopores (3 nm), and macropores up to 252 nm ensure its advanced application with increased mass transfer and mechanical stability.
Knuth *et al.*^84^	Preparation and characterization of graphene nanosheets from graphite flakes through assisted intercalation-expansion using a microwave oven	2024	In this work, the microwave-assisted synthesis of expanded graphite nanosheets is optimized. A mixture of sulfuric and nitric acid intercalation was used for the synthesis. Heat treatment at a temperature as high as 800 °C yielded high-quality GNs with about 1 nm thick, maximum expansion, and minimum thermal degradation. Electrochemical testing by means of techniques such as CV and EIS showed excellent electrochemical properties, therefore underlining this energy-efficient process to produce carbon-based materials of high performance.
Rasuli *et al.*^89^	Microwave-assisted exfoliation and tearing of graphene oxide in the presence of TiO_2_ nanoparticles	2020	The present study is a new way for the reduction, exfoliation, and fragmentation of graphene oxide nanosheets or graphene quantum dots using UV and microwave irradiation in the presence of TiO_2_ nanoparticles. GO sheets that were reduced at 90 W MW power became fragmented at 450 W to ∼20 nm nanosheets. The size reduction is confirmed by broadening photoluminescence and a 3 eV bandgap; the increased functionality is enhanced with the bonding of TiO_2_.

**TABLE IX.** Applications of graphene-based materials in cancer diagnostics, summarizing biosensor designs, targets, and analytical performance metrics.

Author name	Paper Title	Publication Year	Findings/Results
Arshad *et al.*^96^	Applications of graphene-based electrochemical and optical biosensors in early detection of cancer biomarkers	2022	Graphene-based biosensors hold promise in cancer diagnostics due to their high specificity, sensitivity, and selectivity. While the need of the hour is early diagnosis, as in the case of quantifying CA 19–9 in gastrointestinal cancers, with a detection limit as low as 0.006 U/mL, graphene exhibits unique properties and the ability to support multifunctional platforms for simultaneous biomarker detection, making it ideal in early diagnostics of cancer.
Asadi and Ramasamy^105^	Graphene-based Electrochemical Biosensor for Impedimetric Detection of miRNAs as Potential Cancer Biomarkers	2020	Based on graphene, this biosensor is dedicated to the detection of the biomarker miRNA-21, resulting from prostate cancer. A label-free detection with a low detection limit of 3 fM and high selectivity was allowed by the graphene modification of the electrode, providing an excellent performance even in real plasma samples. The sensitivity, stability, and reproducibility will make this proposed biosensor a promising tool for early cancer diagnosis and point-of-care applications.
Lin and Tan^101^	Biosensors for the detection of lung cancer biomarkers: A review on biomarkers, transducing techniques and recent graphene-based implementations	2023	The study have provided the usage of graphene-based biosensors in the detection of lung cancer biomarkers like EGFR, TP53, and CEA. The study employs excellent graphene charge transfer, large surface area, and ease of integration with nanomaterials to enhance performance in biosensors. These biosensors employ optical, electrochemical, and mass-based transduction techniques that enable detection with precision in cancer biomarkers and VOCs, hence advancing early diagnosis in
Novodchuk *et al.*^98^	Graphene-based field effect transistor biosensors for breast cancer detection: A review on biosensing strategies	2021	The study review graphene-based FETs for the biosensing of breast cancer, with the aim of enhancing sensitivity and speed, taking advantage of its good electrical and mechanical properties. Such FET biosensors allow highly precise detection of BC biomarkers with low detection limits while allowing improved mobility and optimization of the “ON/OFF” current ratios. Different device configurations will be explored for developing strategies for signal amplification and improvement that could be put into practice for point-of-care applications.
Pourmadadi *et al.*^97^	Properties and Applications of Graphene and Its Derivatives in Biosensors for Cancer Detection: A Comprehensive Review	2020	The study explores the use of graphene-based biosensors for early cancer detection by targeting biomarkers like DNA, RNA, enzymes, and proteins. Techniques such as field-effect transistors (FETs), fluorescence sensors, SPR biosensors, and electrochemical biosensors leverage graphene's exceptional sensitivity, selectivity, and stability. Derivatives like GO, RGO, and GQDs enhance detection capabilities, offering a wide range of applications despite challenges like scalability and biosafety.

**TABLE X.** Graphene-based biosensors and nanosensors for food safety and contaminant detection, summarizing analytes, transduction principles, and sensitivities.

Author Name	Paper Title	Publication Year	Findings/Results
Li *et al.*^112^	Two-Dimensional Material-Based Electrochemical Sensors/Biosensors for Food Safety and Biomolecular Detection	2024	This study highlights the use of graphene-based and other 2D material biosensors for food safety, focusing on the detection of nitrites, heavy metal ions, antibiotics, and pesticides in food and beverages. Such sensors leverage the structure and chemistry of graphene for enhanced sensitivity and functionalization, therefore culminating in a promising approach toward ensuring both food quality and safety while monitoring health-related small molecules.
Lv *et al.*^115^	Engineering nanomaterials-based biosensors for food safety detection.	2023	This study reviews graphene-based and nanomaterial-enabled biosensors for food safety, addressing contaminants like pathogens, toxins, heavy metals, pesticides, and illegal additives. Overcoming various disadvantages with conventional analytical methods, these technologies stand at the frontiers of enhancing food safety monitoring and therefore represent a fertile ground for interdisciplinary collaboration between food science and nanotechnology.
Neethirajan *et al.*^113^	Nano-biosensor platforms for detecting food allergens – New trends	2021	This study highlights TiO_2_/graphene nanocomposites as advanced photocatalysts for environmental remediation, including air and water purification, self-cleaning surfaces, H_2_ production, and CO_2_ reduction. Graphene may increase the photocatalytic activity of nanomaterials by reducing electron–hole recombination and improving charge transfer. Defect engineering strategies, optimization of crystal facets, and adjustments in the nanomaterial dimensions are underlined to show the maximal environmental applications of nanomaterials under visible light.
Sundramoorthy *et al.*^109^	Graphene-Based Nanosensors and Smart Food Packaging Systems for Food Safety and Quality Monitoring	2019	Graphene-based biosensors are reported herein for the detection of food allergens based on optical, electromechanical, and electrochemical mechanisms. These POC devices help in the prevention of allergic reactions due to the identification of known allergens during the preparation of food. However, growing hypersensitivity, emerging allergens, and lack of detection standards are some of the challenges that reinforce the need to search for innovative sensitive and portable solutions that can enhance the capability of improving management regarding the safety of food.
Bobrinetskiy and Knezevic^106^	Graphene-based biosensors for on-site detection of contaminants in food	2020	This study emphasizes the use of graphene and carbon nanotubes in biosensors for food analysis. The ability for high surface areas, conductivity, and catalysis significantly enhances detection for these chemical contaminants. Nanostructured elements are used as transducers for electrochemical, optical, chemoresistive, and FET biosensors. The development of portable devices for rapid in-field monitoring of food safety is discussed in the review.

**TABLE XI.** Graphene-based biosensor platforms in pharmaceutical and therapeutic monitoring, detailing analytes, sensing strategies, and device performance.

Author Name	Paper Title	Publication Year	Findings/Results
Hemdan *et al.*^125^	Innovations in Biosensor Technologies for Healthcare Diagnostics and Therapeutic Drug Monitoring: Applications, Recent Progress, and Future Research Challenges	2024	This review outlines graphene-based biosensors for pharmaceutical applications, with a focus on disease biomarker detection and therapeutic drug monitoring. Such biosensors allow for a range of precise pharmacokinetic tests, assessment of the effectiveness of specific medications, and real-time follow-up that will improve personalized therapy. The combination of nanotechnology, wearable devices, and miniaturization trends has been found to achieve its transformative power in rapid, sensitive, and portable diagnostic devices in modern medicine.
Kadhim *et al.*^123^	Evaluation of a biosensor-based graphene oxide-DNA nanohybrid for lung cancer	2023	This work presents a GO-DNA nanobiosensor for the detection of lung cancer by mutation analysis. It detects deletion mutations in the EGFR gene through fluorescence quenching and recovery due to DNA hybridization. It opens up a rapid, economical, ultra-sensitive diagnostic platform and proves the potential of GO in detecting the biomarkers of lung cancer through fluorescence spectroscopy.
Redzepi *et al.*^116^	Synthesis of Graphene-Based Biosensors and its Application in Medicine and Pharmacy - A Review	2022	A bilirubin biosensor was developed by immobilizing bilirubin oxidase (BOx) on graphene oxide (GO) and polyaniline (PANI) co-precipitated on ITO glass. Characterized by FTIR, XRD, and Raman spectroscopy, the biosensor showed high selectivity and sensitivity with a rapid response (2 s), low LOD (0.15 nM), and linear detection range (0.01–250 *μ*M). It demonstrated excellent stability, lasting 120 days at 5 °C.
Sabah Ahmed *et al.*^121^	A graphene oxide/polyaniline nanocomposite biosensor: synthesis, characterization, and electrochemical detection of bilirubin	2023	Graphene-based biosensors have shown a high level of sensitivity, excellent biocompatibility, and very low detection limits for different applications in the medical and pharmaceutical areas. Synthesized methods involving graphene and its derivatives in detecting glucose, tumor markers, and drugs by the Hummers' process will lead to earlier diagnosis of diseases and targeted drug delivery. Large surface area and conductivity are some of the properties that make graphene viewed as a transformative material in both biosensing and therapy.
Eksin and Erdem^122^	Fullerene modified single-use electrodes as a convenient biosensor platform for electrochemical monitoring of drug- DNA interaction.	2022	In this work, the development of fullerene-modified pencil graphite electrodes as electrochemical biosensors for investigation into the drug-DNA interaction is reported. Surface characterization was performed by SEM and EIS techniques, showing increased immobilization of dsDNA and sensitivity. The resulting optimized biosensor, ensuring the reproducibility of results, was applied to a study about drug–DNA interactions in a Fe(CN6)4-/3- redox system and was able to ensure a robust platform for essential sensitive electrochemical analysis regarding biomedical applications.

**TABLE XII.** Development of wearable graphene devices for biochemical and physiological monitoring, including sensor configurations and detection limits.

Author Name	Paper Title	Publication Year	Findings/Results
Laliberte *et al.*^131^	A wearable graphene transistor-based biosensor for monitoring IL-6 biomarker	2022	A graphene-based FET-type biosensor is demonstrated for wearable health monitoring in this work. It detects one critical biomarker of the immune response, IL-6, in real time with high sensitivity ranging from 10 pM to 1 nM. Excellent robustness during bending and integration into a battery-powered circuit shows the potential for a standalone wearable biosensor in continuous health monitoring.
Li *et al.*^127^	Flexible enzymatic biosensor based on graphene sponge for glucose detection in human sweat	2023	In this paper, the authors have developed a flexible graphene-based biosensor, named GCGP, for the detection of glucose in sweat, where GS is introduced to enhance its performance. It exhibits high sensitivity. The porous structure of GS allows for efficient enzyme absorption and electron transportation; hence, it might provide excellent performance in wearable devices for the noninvasive monitoring of diabetes during any form of exercise.
Rezaei *et al.*^132^	Wearable Kapton graphene biosensor for detection of toxic gases	2024	a flexible graphene-based biosensor has been developed, which has been embedded in a polyimide film for the noninvasive detection of toxic gases. The paper proposes an RF Planar Resonant Structure sensor to detect dangerous gases such as Fluorine azide, Hydrogen Iodide, Methane, and Carbon monoxide through their distinctive characteristics. Detection by the Graphene-Kapton sensor is very accurate, sensitive, and reliable, hence making it one of the best candidates for wearable safety monitoring devices.
Wang *et al.*^133^	A Wearable and Deformable Graphene-Based Affinity Nanosensor for Monitoring of Cytokines in Biofluids	2020	In this paper, wearable graphene-based field-effect transistor biosensors are developed for the detection of cytokines in biofluids. The biosensor functionalized by aptamer graphene and an ultrathin substrate retains its sensitivity on bending and stretching, with a very low LOD of 2.75 and 2.89 pM for TNF-*α* and IFN-*γ*, respectively. Besides, its nonionic surfactant minimizes nonspecific adsorption, enabling consistent real-time inflammatory marker detection on any part of the non-planar body surface.
Liu *et al.*^94^	Laser-induced graphene (LIG)-driven medical sensors for health monitoring and diseases diagnosis	2022	A variety of applications of LIG in medical sensing, especially in wearable electronics for ion, small molecule, microRNA, protein, and cell detections, are discussed. Considering its rapid and cost-effective fabrication, as well as its good physicochemical properties, this material turns out to be suitable for wearables in physiological sensors that are capable of real-time health monitoring and open new opportunities for diagnostics in advanced medicine.

**TABLE XIII.** Environmental remediation studies using graphene and graphene-based composites, covering adsorption, photocatalysis, and pollutant degradation.

Author Name	Paper Title	Publication Year	Findings/Results
Gao *et al.*^143^	Graphene-based aerogels in water and air treatment: A review	2024	This work highlights the application of graphene-based aerogels in environmental remediation related to the abatement of water and atmospheric pollutants like heavy metal ions, organic dyes, NOx, and VOCs. Their tunable pore size and functional groups will enhance their adsorption/catalysis. The review has underlined their potential use as efficient environmental materials and strategized for their industrial application.
Mishra and Acharya^140^	Recent updates in modification strategies for escalated performance of Graphene/MFe_2_O_4_ heterostructured photocatalysts toward energy and environmental applications	2023	This work presents a review of Graphene/MFe_2_O_4_ heterojunctions for their possible applications in visible-light-driven photocatalytic environmental remediation and energy production. Consequently, this increases its pollutant degradation and separation capabilities due to the combination of electron transport capability from graphene with the magnetic properties of MFe_2_O_4_. Heteratom doping techniques and synthesis of ternary composites further improve the capability of light absorption and rate of reactions, hence showing prospects for GSFs toward sustainable applications using visible light.
Padmanabhan *et al.*^135^	Graphene coupled TiO_2_ photocatalysts for environmental applications: A review	2021	This study highlights TiO_2_/graphene nanocomposites as advanced photocatalysts for environmental remediation, including air and water purification, self-cleaning surfaces, H_2_ production, and CO_2_ reduction. Graphene may increase the photocatalytic activity of nanomaterials by reducing electron–hole recombination and improving charge transfer. Defect engineering strategies, optimization of crystal facets, and adjustments in the nanomaterial dimensions are underlined to show the maximal environmental applications of nanomaterials under visible light.
Wang *et al.*^136^	Environmental Remediation Applications of Carbon Nanotubes and Graphene Oxide: Adsorption and Catalysis	2019	The present review has focused on GO and CNT for environmental remediation serving as adsorbents and catalysts, respectively. CNTs are highly capable of removing organic and inorganic nature pollutants through mechanisms such as *π*–*π* interaction, hydrophobic effect, and electrostatic force. The functionalization of CNTs and GO can enhance or tune their properties to suit requirements for contaminant-specific removals that respond to some challenges in wastewater treatments.
Yin *et al.*^142^	The Application of Carbon Nanotube/Graphene-Based Nanomaterials in Wastewater Treatment	2020	This review covers the applications of CNT- and graphene-based nanomaterials for organic wastewater treatment. Because of their large surface area, highly tunable surface properties, and chemical stability, they can absorb effectively in extreme conditions. Key focuses are given to adsorption mechanisms, surface modification to enhance the performance, engineering challenges associated with stability, kinetics, and reversibility, and economic considerations toward scalable implementation in real wastewater systems.

## References

[1] S. Szunerits and R. Boukherroub, “Graphene-based biosensors,” Interface Focus 8(3), 20160132 (2018). 10.1098/rsfs.2016.0132 29696084 PMC5915654

[2] H. A. Alhazmi *et al.*, “Graphene-based biosensors for disease theranostics: Development, applications, and recent advancements,” Nanotechnol. Rev. 11(1), 96–116 (2021). 10.1515/ntrev-2022-0009

[3] F. H. Abrha *et al.*, “Graphene-based biosensors for detecting coronavirus: A brief review,” Nanoscale 15, 18184 (2023). 10.1039/D3NR04583H 37927083

[4] O. Oshin *et al.*, “Graphene-based biosensor for early detection of iron deficiency,” Sensors 20(13), 3688 (2020). 10.3390/s20133688 32630192 PMC7374411

[5] M. Kujawska *et al.*, “Using graphene-based biosensors to detect dopamine for efficient Parkinson's disease diagnostics,” Biosensors 11(11), 433 (2021). 10.3390/bios11110433 34821649 PMC8615362

[6] C. Deepa, L. Rajeshkumar, and M. Ramesh, “Preparation, synthesis, properties and characterization of graphene-based 2D nano-materials for biosensors and bioelectronics,” J. Mater. Res. Technol. 19, 2657–2694 (2022). 10.1016/j.jmrt.2022.06.023

[7] B. Karki *et al.*, “Hemoglobin detection in blood samples using a graphene-based surface plasmon biosensor,” Optik 270, 169947 (2022). 10.1016/j.ijleo.2022.169947

[8] P. R. de Almeida III *et al.*, “Development of a graphene-based biosensor for detecting recombinant cyanovirin-N,” Biosensors 10(12), 206 (2020). 10.3390/bios10120206 33339087 PMC7765543

[9] R. Goldoni *et al.*, “Recent advances in graphene-based nanobiosensors for salivary biomarker detection,” Biosens. Bioelectron. 171, 112723 (2021). 10.1016/j.bios.2020.112723 33096432 PMC7666013

[10] S. Szunerits *et al.*, “Graphene-based field-effect transistors for biosensing: Where is the field heading to?” Anal. BioanalChem. 416(9), 2137–2150 (2024). 10.1007/s00216-023-04760-1 PMC10239049 37269306

[11] J. Monkrathok *et al.*, “Enhancing glucose biosensing with graphene oxide and ferrocene-modified linear poly(ethylenimine),” Biosensors 14(4), 161 (2024). 10.3390/bios14040161 38667154 PMC11048651

[12] A. Battisti, S. K. Samal, and D. Puppi, “Biosensing systems based on graphene oxide fluorescence quenching effect,” Micromachines 14(8), 1522 (2023). 10.3390/mi14081522 37630058 PMC10456591

[13] F. Fauzi *et al.*, “Gas and humidity sensing with quartz crystal microbalance (QCM) coated with graphene-based materials–A mini review,” Sens. Actuators, A 330, 112837 (2021). 10.1016/j.sna.2021.112837

[14] F. Gao *et al.*, “Wearable and flexible electrochemical sensors for sweat analysis: A review,” Microsyst. Nanoeng. 9(1), 1 (2023). 10.1038/s41378-022-00443-6 36597511 PMC9805458

[15] M. G. Sumdani *et al.*, “Recent advances of the graphite exfoliation processes and structural modification of graphene: A review,” J. Nanopart. Res. 23, 253 (2021). 10.1007/s11051-021-05371-6

[16] M. A. Baqiya *et al.*, “Structural study on graphene-based particles prepared from old coconut shell by acid–assisted mechanical exfoliation,” Adv. Powder Technol. 31, 2072–2078 (2020). 10.1016/j.apt.2020.02.039

[17] Y. Raza *et al.*, “Production and investigation of mechanical properties of graphene/polystyrene nano composites,” J. Polym. Res. 28, 217 (2021). 10.1007/s10965-021-02560-8

[18] A. A. Pirzado *et al.*, “Few-layer graphene from mechanical exfoliation of graphite-based materials: Structure-dependent characteristics,” ChemEngineering 3, 37 (2019). 10.3390/chemengineering3020037

[19] C. Cheng *et al.*, “Tandem chemical modification/mechanical exfoliation of graphite: Scalable synthesis of high-quality, surface-functionalized graphene,” Carbon 145, 668 (2019). 10.1016/j.carbon.2019.01.079

[20] A. Saloum, B. Al-Tamimi, and S. B. H. Farid, “Preparation of graphene nanosheets from graphite flakes via shear assisted exfoliation,” Eng. Technol. J. 39, 1663 (2021). 10.30684/etj.v39i11.2219

[21] E. Cuadros-Lugo *et al.*, “Removal of MgO impurity crystals by mechanical milling exfoliation of graphene obtained by CO_2_ atmosphere synthesis method.,” Microsc. Microanal. 27, 1762–1764 (2021). 10.1017/S1431927621006450

[22] L. A. Myroniuk *et al*., “Mechanical exfoliation of graphite to graphene in polyvinylpyrrolidone aqueous solution,” Him. Fiz. Tehnol. Poverhni 14, 230–236 (2023). 10.15407/hftp14.02.230

[23] P. Dash, T. K. Rout, and S. K. Biswal, “Study on the preparation of GO and RGO by chemical and mechanical exfoliation of natural graphite for the aluminum industry,” J. Sustainable Metall. 6, 26–33 (2020). 10.1007/s40831-019-00251-9

[24] J. Fan *et al.*, “Improved exfoliation of surface-functionalized graphene oxide by epoxy monomer and enhanced mechanical properties of epoxy nanocomposites,” J. Mater. Sci. 57, 366–382 (2022). 10.1007/s10853-021-06580-z

[25] Z. Chen *et al.*, “Effects of hybrid surfactants on the quality and yield of graphene using a novel electrochemical-mechanical exfoliation process,” Int. J. Adv. Manuf. Technol. 119, 6809–6817 (2022). 10.1007/s00170-021-08562-6

[26] Y. Zhang *et al.*, “Mechanical exfoliation assisted with carbon nanospheres to prepare a few-layer graphene for flexible strain sensor,” Appl. Surf. Sci. 611, 155649 (2023). 10.1016/j.apsusc.2022.155649

[27] M. A. Rahman, M. Salam, and A. Ashraf, “In-situ shear exfoliation of graphene from graphite polymer nanocomposites for lung and heart motion,” in *ASME 2023 International Mechanical Engineering Congress and Exposition*, Volume 12: Micro- and Nano-Systems Engineering and Packaging (ASME, 2023).

[28] D. Chow *et al.*, “Mechanical exfoliation of expanded graphite to graphene-based materials and modification with palladium nanoparticles for hydrogen storage,” Nanomaterials 13, 2588 (2023). 10.3390/nano13182588 37764617 PMC10534434

[29] A. F. Kurniawan *et al.*, “Mechanical exfoliation of reduced graphene oxide from old coconut shell as radar absorber in X-band,” Mater. Sci. Forum 966, 25–29 (2019). 10.4028/www.scientific.net/MSF.966.25

[30] Z. Li *et al.*, “Mechanisms of liquid-phase exfoliation for the production of graphene,” ACS Nano 14(9), 10976–10985 (2020). 10.1021/acsnano.0c03916 32598132

[31] A. Dimiev *et al.*, “Layer-by-layer removal of graphene for device patterning,” Science 331, 6021 (2011). 10.1126/science.1199183 21385709

[32] A. Kaur *et al.*, “Dual frequency ultrasonic liquid phase exfoliation method for the production of few layer graphene in green solvents,” Ultrason. Sonochem. 108, 106954 (2024). 10.1016/j.ultsonch.2024.106954 38879962 PMC11211887

[33] M. Telkhozhayeva *et al.*, “Higher ultrasonic frequency liquid phase exfoliation leads to larger and monolayer to few-layer flakes of 2D layered materials,” Langmuir 37(15), 4504–4514 (2021). 10.1021/acs.langmuir.0c03668 33724843

[34] H. Chacham *et al.*, “Controlling the morphology of nanoflakes obtained by liquid-phase exfoliation: Implications for the mass production of 2D materials,” ACS Appl. Nano Mater. 3(12), 12095–12105 (2020). 10.1021/acsanm.0c02598

[35] C. V. Gomez *et al.*, “The liquid exfoliation of graphene in polar solvents,” Appl. Surf. Sci. 546, 149046 (2021). 10.1016/j.apsusc.2021.149046

[36] H. Ma and Z. Shen, “Exfoliation of graphene nanosheets in aqueous media,” Ceram. Int. 46(14), 21873–21887 (2020). 10.1016/j.ceramint.2020.05.314

[37] Ö. Güler *et al.*, “The production of graphene by direct liquid phase exfoliation of graphite at moderate sonication power by using low boiling liquid media: The effect of liquid media on yield and optimization,” Ceram. Int. 47(1), 521–533 (2021). 10.1016/j.ceramint.2020.08.159

[38] A. A. Moosa and M. Abed, “Graphene preparation and graphite exfoliation,” Turk. J. Chem. 45(3), 493–519 (2021). 10.3906/kim-2101-19 34385847 PMC8326494

[39] X. Han *et al.*, “Interfacial interaction and steric repulsion in polymer-assisted liquid exfoliation to produce high-quality graphene,” Chem. Pap. 74, 757–765 (2020). 10.1007/s11696-019-00928-1

[40] V. Paolucci *et al.*, “Sustainable liquid-phase exfoliation of layered materials with nontoxic polarclean solvent,” ACS Sustainable Chem. Eng. 8(51), 18830–18840 (2020). 10.1021/acssuschemeng.0c04191 33828931 PMC8018326

[41] A. Islam *et al.*, “Ultra-fast, chemical-free, mass production of high quality exfoliated graphene,” ACS Nano 15(1), 1775–1784 (2021). 10.1021/acsnano.0c09451 33448799

[42] F. I. Alzakia and S. C. Tan, “Liquid-exfoliated 2D materials for optoelectronic applications,” Adv. Sci. 8(11), 2003864 (2021). 10.1002/advs.202003864 PMC8188210 34105282

[43] Y. Liu and R. Li, “Study on ultrasound-assisted liquid-phase exfoliation for preparing graphene-like molybdenum disulfide nanosheets,” Ultrason. Sonochem. 63, 104923 (2020). 10.1016/j.ultsonch.2019.104923 31945553

[44] Y. He *et al.*, “Biocompatible 2D materials via liquid phase exfoliation,” Adv. Mater. 36, 2310999 (2024). 10.1002/adma.202310999 38457626

[45] S. Bhowmik and A. G. Rajan, “Chemical vapor deposition of 2D materials: A review of modeling, simulation, and machine learning studies,” iScience 25(3), 103832 (2022). 10.1016/j.isci.2022.103832 35243221 PMC8857588

[46] J. Wang *et al.*, “A review of graphene synthesis at low temperatures by CVD methods,” Carbon 171, 980–980 (2021). 10.1016/j.carbon.2020.05.089

[47] Y. Song *et al.*, “Graphene transfer: Paving the road for applications of chemical vapor deposition graphene,” Small 17(48), 2007600 (2021). 10.1002/smll.202007600 33661572

[48] Y. Cheng, K. Wang, Y. Qi, and Z Liu, “Chemical Vapor Deposition Method for Graphene Fiber Materials,” Acta Physico Chimica Sinica 38(2), 2006046 (2022). 10.3866/PKU.WHXB202006046

[49] M. Saeed *et al.*, “Chemical vapour deposition of graphene—Synthesis, characterisation, and applications: A review,” Molecules 25(17), 3856 (2020). 10.3390/molecules25173856 32854226 PMC7503287

[50] K. R. Nandanapalli *et al.*, “User-friendly methodology for chemical vapor deposition–grown graphene-layers transfer: Design and implementation,” Mater. Today Chem. 21, 100546 (2021). 10.1016/j.mtchem.2021.100546

[51] K. Jia *et al.*, “Superclean growth of graphene using a cold-wall chemical vapor deposition approach,” Angew. Chem. 132(39), 17367–17371 (2020). 10.1002/ange.202005406 32542959

[52] Y. Zhang *et al.*, “Role of hydrogen and oxygen in the study of substrate surface impurities and defects in the chemical vapor deposition of graphene,” Carbon 185, 82–95 (2021). 10.1016/j.carbon.2021.09.016

[53] J. Su and C. Li, “Effect of plasma-enhanced chemical vapor deposition (PECVD) graphene content on the properties of EPDM/graphene composites,” J. Mater. Sci.: Mater. Electron. 32(7), 9065–9073 (2021). 10.1007/s10854-021-05575-5

[54] H. Wang *et al.*, “Large-scale chemical vapor deposition synthesis of graphene nanoribbions/carbon nanotubes composite for enhanced membrane capacitive deionization,” J. Electroanal. Chem. 904, 115907 (2022). 10.1016/j.jelechem.2021.115907

[55] B. Sun *et al.*, “Synthesis of wafer-scale graphene with chemical vapor deposition for electronic device applications,” Adv. Mater. Technol. 6(7), 2000744 (2021). 10.1002/admt.202000744

[56] S. Pezzini *et al.*, “30-twisted bilayer graphene quasicrystals from chemical vapor deposition.,” Nano Lett. 20(5), 3313–3319 (2020). 10.1021/acs.nanolett.0c00172 32297749

[57] S. Xu *et al.*, “Chemical vapor deposition of graphene on thin-metal films,” Cell Rep. Phys. Sci. 2(3), 100372 (2021). 10.1016/j.xcrp.2021.100372

[58] H. Mishra *et al.*, “Experimental advances in charge and spin transport in chemical vapor deposited graphene,” J. Phys. Mater. 4(4), 042007 (2021). 10.1088/2515-7639/ac1247

[59] M. Shekhirev *et al.*, “Highly selective gas sensors based on graphene nanoribbons grown by chemical vapor deposition,” ACS Appl. Mater. Interfaces 12(6), 7392–7402 (2020). 10.1021/acsami.9b13946 32011111

[60] B. Ali *et al.*, “Synthesis and characterization of high-quality multi layered graphene by electrochemical exfoliation of graphite,” Res. Eng. Struct. Mater. 8(3), 447–462 (2022). 10.17515/resm2022.384na0121

[61] R. Srivastava and P. K. Singh, “Fabrication & characterizations of reduced graphene oxide via low potential electrochemical exfoliation followed by thermal treatment,” Mater. Today: Proc. (published online) (2023). 10.1016/j.matpr.2023.02.120

[62] H. Aghamohammadi *et al.*, “Recent advances in one-pot functionalization of graphene using electrochemical exfoliation of graphite: A review study,” Synth. Met. 269, 116549 (2020). 10.1016/j.synthmet.2020.116549

[63] G. Bilgiç, “Reduced graphene synthesis via eco-friendly electrochemical exfoliation method,” Kastamonu Univ. J. Eng. Sci. 10(1), 38–43 (2024). 10.55385/kastamonujes.1477345

[64] J. R. Prekodravac *et al.*, “A comprehensive review on selected graphene synthesis methods: From electrochemical exfoliation through rapid thermal annealing towards biomass pyrolysis,” J. Mater. Chem. C 9(21), 6722–6748 (2021). 10.1039/D1TC01316E

[65] A. Loudiki *et al.*, “Preparation of graphene samples via electrochemical exfoliation of pencil electrode: Physico-electrochemical characterization,” Appl. Surf. Sci. Adv. 7, 100195 (2022). 10.1016/j.apsadv.2021.100195

[66] Z. Ji *et al.*, “A structured catalyst support combining electrochemically exfoliated graphene oxide and carbon black for enhanced performance and durability in low-temperature hydrogen fuel cells,” Energy 226, 120318 (2021). 10.1016/j.energy.2021.120318

[67] W. H. Danial *et al.*, “A short review on electrochemical exfoliation of graphene and graphene quantum dots,” Carbon Lett. 31, 371–388 (2021). 10.1007/s42823-020-00212-3

[68] L. Li *et al.*, “Preparation and application of graphene-based hybrid materials through electrochemical exfoliation,” J. Electrochem. Soc. 167(8), 086511 (2020). 10.1149/1945-7111/ab933b

[69] H. Lee *et al.*, “Role of anions on electrochemical exfoliation of graphite into graphene in aqueous acids,” Carbon 167, 816–825 (2020). 10.1016/j.carbon.2020.06.044

[70] J. Chen *et al.*, “One step electrochemical exfoliation of natural graphite flakes into graphene oxide for polybenzimidazole composite membranes giving enhanced performance in high temperature fuel cells,” J. Power Sources 491, 229550 (2021). 10.1016/j.jpowsour.2021.229550

[71] N. Kumar, M. A. Sadique, and R. Khan, “Electrochemical exfoliation of graphene quantum dots from waste dry cell battery for biosensor applications,” Mater. Lett. 305, 130829 (2021). 10.1016/j.matlet.2021.130829

[72] B. Liu *et al.*, “Electrochemically exfoliated chlorine-doped graphene for flexible all-solid-state micro-supercapacitors with high volumetric energy density,” Adv. Mater. 34(19), 2106309 (2022). 10.1002/adma.202106309 35263463

[73] A. D. Pingale *et al.*, “Facile synthesis of graphene by ultrasonic-assisted electrochemical exfoliation of graphite,” Mater. Today: Proc. 44, 467–472 (2021). 10.1016/j.matpr.2020.10.045

[74] X. Zhao *et al.*, “Electrochemical exfoliation of graphene as an anode material for ultra-long cycle lithium ion batteries,” J. Phys. Chem. Solids 139, 109301 (2020). 10.1016/j.jpcs.2019.109301

[75] J. Wu *et al.*, “Efficient preparation of high-quality graphene via anodic and cathodic simultaneous electrochemical exfoliation under the assistance of microwave,” J. Colloid Interface Sci. 608, 1422–1431 (2022). 10.1016/j.jcis.2021.10.098 34742062

[76] S. Rattan, S. Kumar, and J. Goswamy, “Microwave-aided exfoliation and reduction of graphene oxide,” J. Nanosci. Nanotechnol. 21(3), 1667–1671 (2021). 10.1166/jnn.2021.19022 33404431

[77] R. Jakhar, J. E. Yap, and R. Joshi, “Microwave reduction of graphene oxide,” Carbon 170, 277–293 (2020). 10.1016/j.carbon.2020.08.034

[78] B. Gürünlü, Ç. Taşdelen-Yücedağ, and M. Bayramoğlu, “One pot synthesis of graphene through microwave assisted liquid exfoliation of graphite in different solvents,” Molecules 27(15), 5027 (2022). 10.3390/molecules27155027 35956975 PMC9370801

[79] J. Wu *et al.*, “Rapid microwave-assisted bulk production of high-quality reduced graphene oxide for lithium ion batteries,” Materialia 13, 100833 (2020). 10.1016/j.mtla.2020.100833

[80] R. Kumar *et al.*, “A review on the current research on microwave processing techniques applied to graphene-based supercapacitor electrodes: An emerging approach beyond conventional heating,” J. Energy Chem. 74, 252–282 (2022). 10.1016/j.jechem.2022.06.051

[81] J. Wang *et al.*, “Recent progress in microwave-assisted preparations of 2D materials and catalysis applications,” Nanotechnology 33(34), 342002 (2022). 10.1088/1361-6528/ac6c97 35508114

[82] M. Skorupska, A. Ilnicka, and J. P. Lukaszewicz, “N-doped graphene foam obtained by microwave-assisted exfoliation of graphite,” Sci. Rep. 11(1), 2044 (2021). 10.1038/s41598-021-81769-5 33479478 PMC7820460

[83] Y. Yang, Z. Wang, and S. Zheng, “Secondary exfoliation of electrolytic graphene oxide by ultrasound assisted microwave technique,” Nanomaterials 12(1), 68 (2021). 10.3390/nano12010068 35010018 PMC8746382

[84] R. D. Knuth *et al.*, “Preparation and characterization of graphene nanosheets from graphite flakes through assisted intercalation-expansion using a microwave oven,” J. Solid State Electrochem. 28(7), 2059–2070 (2024). 10.1007/s10008-023-05736-y

[85] Q. Ji *et al.*, “Scalable fabrication of holey graphene nanosheets by electrochemical intercalation and microwave-assisted expansion of graphite,” Appl. Surf. Sci. 560, 150052 (2021). 10.1016/j.apsusc.2021.150052

[86] A. Hamra *et al.*, “Microwave exfoliated graphene-based materials for flexible solid-state supercapacitor,” J. Mol. Struct. 1220, 128710 (2020). 10.1016/j.molstruc.2020.128710

[87] R. Kumar *et al.*, “Microwave-assisted thin reduced graphene oxide-cobalt oxide nanoparticles as hybrids for electrode materials in supercapacitor,” J. Energy Storage 40, 102724 (2021). 10.1016/j.est.2021.102724

[88] S. K. Kandasamy *et al.*, “Microwave-assisted graphene-based conducting polymer materials for supercapacitors,” in *Handbook of Supercapacitor Materials: Synthesis, Characterization, and Applications* (Wiley, 2021), pp. 299–326.

[89] H. Rasuli *et al.*, “Microwave-assisted exfoliation and tearing of graphene oxide in the presence of TiO_2_ nanoparticles,” Results Phys. 18, 103200 (2020). 10.1016/j.rinp.2020.103200

[90] J. Lin *et al.*, “Laser-induced porous graphene films from commercial polymers,” Nat. Commun. 5(1), 5714 (2014). 10.1038/ncomms6714 25493446 PMC4264682

[91] K. Kim *et al.*, “Recent advances in sensitive surface-enhanced Raman scattering-based lateral flow assay platforms for point-of-care diagnostics of infectious diseases,” Sens. Actuators, B 329, 129214 (2021). 10.1016/j.snb.2020.129214 PMC9759493 36568647

[92] H. Moon and B. Ryu, “Review of laser-induced graphene (LIG) produced on eco-friendly substrates,” Int. J. Precis. Eng. Manuf.-Green. Technol. 11(4), 1279–1294 (2024). 10.1007/s40684-024-00595-y

[93] Z. Zhang *et al.*, “A review of laser-induced graphene: From experimental and theoretical fabrication processes to emerging applications,” Carbon 214, 118356 (2023). 10.1016/j.carbon.2023.118356

[94] J. Liu *et al.*, “Laser-induced graphene (LIG)-driven medical sensors for health monitoring and diseases diagnosis,” Microchim. Acta 189, 1–14 (2022). 10.1007/s00604-021-05157-6 PMC8743164 35001163

[95] V. P. Wanjari *et al.*, “Laser-induced graphene-based electrochemical biosensors for environmental applications: A perspective,” Environ. Sci. Pollut. Res. 30(15), 42643–42657 (2022). 10.1007/s11356-022-21035-x 35622288

[96] F. Arshad *et al.*, “Applications of graphene-based electrochemical and optical biosensors in early detection of cancer biomarkers,” Colloids Surf. B 212, 112356 (2022). 10.1016/j.colsurfb.2022.112356 35123193

[97] M. Pourmadadi *et al.*, “Properties and applications of graphene and its derivatives in biosensors for cancer detection: A comprehensive review,” Biosensors 12(5), 269 (2022). 10.3390/bios12050269 35624570 PMC9138779

[98] I. Novodchuk, M. Bajcsy, and M. Yavuz, “Graphene-based field effect transistor biosensors for breast cancer detection: A review on biosensing strategies,” Carbon 172, 431–453 (2021). 10.1016/j.carbon.2020.10.048

[99] A. Mohammadpour-Haratbar *et al.*, “Graphene-based electrochemical biosensors for breast cancer detection,” Biosensors 13(1), 80 (2023). 10.3390/bios13010080 36671915 PMC9855997

[100] Y. Bai, T. Xu, and X. Zhang, “Graphene-based biosensors for detection of biomarkers,” Micromachines 11(1), 60 (2020). 10.3390/mi11010060 31947894 PMC7019259

[101] L. P. Lin and M. T. T. Tan, “Biosensors for the detection of lung cancer biomarkers: A review on biomarkers, transducing techniques and recent graphene-based implementations,” Biosens. Bioelectron. 237, 115492 (2023). 10.1016/j.bios.2023.115492 37421797

[102] D. Ozkan-Ariksoysal, “Current perspectives in graphene oxide-based electrochemical biosensors for cancer diagnostics,” Biosensors 12(8), 607 (2022). 10.3390/bios12080607 36005004 PMC9405788

[103] J. Sengupta and C. M. Hussain, “CNT and graphene-based transistor biosensors for cancer detection: A review,” Biomolecules 13(7), 1024 (2023). 10.3390/biom13071024 37509060 PMC10377131

[104] M. Safari *et al.*, “Carbon-based biosensors from graphene family to carbon dots: A viewpoint in cancer detection,” Talanta 258, 124399 (2023). 10.1016/j.talanta.2023.124399 36870153

[105] H. Asadi and R. P. Ramasamy, “Graphene-based electrochemical biosensor for impedimetric detection of miRNAs as potential cancer biomarkers,” J. Electrochem. Soc. 167(16), 167523 (2020). 10.1149/1945-7111/abd284

[106] I. I. Bobrinetskiy and N. Z. Knezevic, “Graphene-based biosensors for on-site detection of contaminants in food,” Anal. Methods 10(42), 5061–5070 (2018). 10.1039/C8AY01913D

[107] W. K. Abdelbasset *et al.*, “Comparison and evaluation of the performance of graphene-based biosensors.,” Carbon Lett. 32(4), 927 (2022). 10.1007/s42823-022-00338-6

[108] J. Peña-Bahamonde *et al.*, “Recent advances in graphene-based biosensor technology with applications in life sciences,” J. Nanobiotechnol. 16(1), 75 (2018). 10.1186/s12951-018-0400-z PMC6150956 30243292

[109] A. K. Sundramoorthy, T. H. V. Kumar, and S. Gunasekaran, “Graphene-based nanosensors and smart food packaging systems for food safety and quality monitoring,” in *Graphene Bioelectronics* (Elsevier, 2018), pp. 267–306.

[110] X. Hou *et al.*, “Recent developments in three-dimensional graphene-based electrochemical sensors for food analysis,” Trends Food Sci. Technol. 105, 76–92 (2020). 10.1016/j.tifs.2020.09.004

[111] S. Taniselass, M. M. Arshad, and S. C. Gopinath, “Graphene-based electrochemical biosensors for monitoring noncommunicable disease biomarkers,” Biosens. Bioelectron. 130, 276–292 (2019). 10.1016/j.bios.2019.01.047 30771717

[112] T. Li *et al.*, “Two-dimensional material-based electrochemical sensors/biosensors for food safety and biomolecular detection,” Biosensors 12(5), 314 (2022). 10.3390/bios12050314 35624615 PMC9138342

[113] S. Neethirajan *et al.*, “Nano-biosensor platforms for detecting food allergens–New trends,” Sens. Bio-Sens. Res. 18, 13–30 (2018). 10.1016/j.sbsr.2018.02.005

[114] S. Shahriari *et al.*, “Graphene and graphene oxide as a support for biomolecules in the development of biosensors,” Nanotechnol. Sci. Appl. 14, 197–220 (2021). 10.2147/NSA.S334487 34815666 PMC8605898

[115] M. Lv *et al.*, “Engineering nanomaterials-based biosensors for food safety detection,” Biosens. Bioelectron. 106, 122–128 (2018). 10.1016/j.bios.2018.01.049 29414078

[116] S. Redzepi, D. Mulic, and M. Dedic, “Synthesis of graphene-based biosensors and its application in medicine and pharmacy-A review,” Medicon Med. Sci. 2(1), 35–45 (2022).

[117] R. Sharma, “Therapeutic voyage of graphene-based biosensor,” Lett. Drug Des. Discovery 21(10), 1662–1674 (2024). 10.2174/0115701808291102240130113741

[118] S. Savita *et al.*, “Brief review on the synthesis, cytotoxixity, bioavailability and various applications of graphene nanomaterials,” Scholars Acad. J. Pharm. (published online) (2020). 10.36347/sajp.2020.v09i09.004

[119] I. Kulakova and G. Lisichkin, “Potential directions in the use of graphene nanomaterials in pharmacology and biomedicine,” Pharm. Chem. J. 56(1), 1–11 (2022). 10.1007/s11094-022-02594-2

[120] A. K. Singh *et al.*, “Optical biosensors: A decade in review,” Alexandria Eng. J. 67, 673–691 (2023). 10.1016/j.aej.2022.12.040

[121] N. Sabah Ahmed *et al.*, “A graphene oxide/polyaniline nanocomposite biosensor: Synthesis, characterization, and electrochemical detection of bilirubin,” RSC Adv. 13(51), 36280–36292 (2023). 10.1039/D3RA06815C 38090067 PMC10714673

[122] E. Eksin and A. Erdem, “Fullerene modified single-use electrodes as a convenient biosensor platform for electrochemical monitoring of drug-DNA interaction,” J. Res. Pharm. 26(4), 997 (2022). 10.29228/jrp.197

[123] M. M. Kadhim *et al.*, “Evaluation of a biosensor-based graphene oxide-DNA nanohybrid for lung cancer,” RSC Adv. 13(4), 2487–2500 (2023). 10.1039/D2RA05808A 36741187 PMC9843741

[124] C. Laghlimi *et al.*, “Recent advances in electrochemical sensors and biosensors for monitoring drugs and metabolites in pharmaceutical and biological samples,” ADMET DMPK 11(2), 151–173 (2023). 10.5599/admet.1709 37325116 PMC10262219

[125] M. Hemdan *et al.*, “Innovations in biosensor technologies for healthcare diagnostics and therapeutic drug monitoring: Applications, recent progress, and future research challenges,” Sensors 24(16), 5143 (2024). 10.3390/s24165143 39204840 PMC11360123

[126] B. Hu *et al.*, “Advances in flexible graphene field-effect transistors for biomolecule sensing,” Front. Bioeng. Biotechnol. 11, 1218024 (2023). 10.3389/fbioe.2023.1218024 37485314 PMC10361656

[127] B. Li *et al.*, “Flexible enzymatic biosensor based on graphene sponge for glucose detection in human sweat,” Surf. Interfaces 36, 102525 (2023). 10.1016/j.surfin.2022.102525

[128] A. Qureshi and J. H. Niazi, “Graphene-interfaced flexible and stretchable micro–nano electrodes: From fabrication to sweat glucose detection,” Mater. Horiz. 10(5), 1580–1607 (2023). 10.1039/D2MH01517J 36880340

[129] H. Zhang *et al.*, “Graphene-enabled wearable sensors for healthcare monitoring,” Biosens. Bioelectron. 197, 113777 (2022). 10.1016/j.bios.2021.113777 34781177

[130] S. U. Singh *et al.*, “Advanced wearable biosensors for the detection of body fluids and exhaled breath by graphene,” Microchim. Acta 189(6), 236 (2022). 10.1007/s00604-022-05317-2 PMC9146825 35633385

[131] K. E. Laliberte *et al.*, “A wearable graphene transistor-based biosensor for monitoring IL-6 biomarker,” Microelectron. Eng. 262, 111835 (2022). 10.1016/j.mee.2022.111835

[132] I. Rezaei *et al.*, “Wearable Kapton graphene biosensor for detection of toxic gases,” J. Hazard. Mater. Adv. 15, 100452 (2024). 10.1016/j.hazadv.2024.100452

[133] Z. Wang *et al.*, “A wearable and deformable graphene-based affinity nanosensor for monitoring of cytokines in biofluids,” Nanomaterials 10(8), 1503 (2020). 10.3390/nano10081503 32751815 PMC7466379

[134] Y. Song *et al.*, “Self-powered wearable biosensors,” Acc. Mater. Res. 2(3), 184–197 (2021). 10.1021/accountsmr.1c00002

[135] N. T. Padmanabhan *et al.*, “Graphene coupled TiO_2_ photocatalysts for environmental applications: A review,” Chemosphere 271, 129506 (2021). 10.1016/j.chemosphere.2020.129506 33445017

[136] Y. Wang *et al.*, “Environmental remediation applications of carbon nanotubes and graphene oxide: Adsorption and catalysis,” Nanomaterials 9(3), 439 (2019). 10.3390/nano9030439 30875970 PMC6474092

[137] H. Chaudhuri and Y.-S. Yun, “Synthesis and environmental applications of graphene oxide/layered double hydroxides and graphene oxide/MXenes: A critical review,” Sep. Purif. Technol. 297, 121518 (2022). 10.1016/j.seppur.2022.121518

[138] C. H. A. Tsang *et al.*, “Graphene materials in green energy applications: Recent development and future perspective,” Renewable Sustainable Energy Rev. 120, 109656 (2020). 10.1016/j.rser.2019.109656

[139] M. H. Facure *et al.*, “A review on graphene quantum dots and their nanocomposites: From laboratory synthesis towards agricultural and environmental applications.,” Environ. Sci. Nano 7(12), 3710–3734 (2020). 10.1039/D0EN00787K

[140] S. Mishra and R. Acharya, “Recent updates in modification strategies for escalated performance of Graphene/MFe_2_O_4_ heterostructured photocatalysts towards energy and environmental applications,” J. Alloys Compd. 960, 170576 (2023). 10.1016/j.jallcom.2023.170576

[141] M. Li *et al.*, “Graphene and graphene-based nanocomposites used for antibiotics removal in water treatment: A review,” Chemosphere 226, 360–380 (2019). 10.1016/j.chemosphere.2019.03.117 30947046

[142] Z. Yin *et al.*, “The application of carbon nanotube/graphene-based nanomaterials in wastewater treatment,” Small 16(15), 1902301 (2020). 10.1002/smll.201902301 31788946

[143] B. Gao *et al.*, “Graphene-based aerogels in water and air treatment: A review,” Chem. Eng. J. 484, 149604 (2024). 10.1016/j.cej.2024.149604

[144] M. F. Hossain, N. Akther, and Y. Zhou, “Recent advancements in graphene adsorbents for wastewater treatment: Current status and challenges,” Chin. Chem. Lett. 31(10), 2525–2538 (2020). 10.1016/j.cclet.2020.05.011

[145] D. L. Silva *et al.*, “Raman spectroscopy analysis of number of layers in mass-produced graphene flakes,” Carbon 161, 181–189 (2020). 10.1016/j.carbon.2020.01.050

[146] S. Farah *et al.*, “Comparison of thermally and chemically reduced graphene oxides by thermal analysis and Raman spectroscopy,” J. Therm. Anal. Calorim. 142, 331–337 (2020). 10.1007/s10973-020-09719-3

[147] Z. Li *et al.*, “Raman spectroscopy of carbon materials and their composites: Graphene, nanotubes and fibres,” Prog. Mater. Sci. 135, 101089 (2023). 10.1016/j.pmatsci.2023.101089

[148] M. V. Moutinho, P. Venezuela, and M. A. Pimenta, “Raman spectroscopy of twisted bilayer graphene,” C 7(1), 10 (2021). 10.3390/c7010010 PMC8390699 34446790

[149] V. Scardaci and G. Compagnini, “Raman spectroscopy investigation of graphene oxide reduction by laser scribing,” C 7(2), 48 (2021). 10.3390/c7020048 PMC8385394 34466636

[150] A. Jorio and R. Saito, “Raman spectroscopy for carbon nanotube applications,” J. Appl. Phys. 129(2), 021102 (2021). 10.1063/5.0030809

[151] Y.-C. Leng *et al.*, “Zenith-angle resolved polarized Raman spectroscopy of graphene,” Carbon 191, 471–476 (2022). 10.1016/j.carbon.2022.02.012

[152] L. M. Malard *et al.*, “Raman spectroscopy in graphene,” Phys. Rep. 473(5–6), 51–87 (2009). 10.1016/j.physrep.2009.02.003

[153] J.-B. Wu *et al.*, “Raman spectroscopy of graphene-based materials and its applications in related devices,” Chem. Soc. Rev. 47(5), 1822–1873 (2018). 10.1039/C6CS00915H 29368764

[154] C. Casiraghi *et al.*, “Raman spectroscopy of graphene edges,” Nano Lett. 9(4), 1433–1441 (2009). 10.1021/nl8032697 19290608

[155] A. M. Brańczyk, D. B. Turner, and G. D. Scholes, “Crossing disciplines-A view on two-dimensional optical spectroscopy,” Ann. Phys. 526(1–2), 31–49 (2014). 10.1002/andp.201300153

[156] L. A. Falkovsky, “Optical properties of graphene,” J. Phys.: Conf. Ser. 129, 012004 (2008). 10.1088/1742-6596/129/1/012004

[157] K. F. Mak *et al.*, “Optical spectroscopy of graphene: From the far infrared to the ultraviolet,” Solid State Commun. 152(15), 1341–1349 (2012). 10.1016/j.ssc.2012.04.064

[158] H. Riesen, C. Wiebeler, and S. Schumacher, “Optical spectroscopy of graphene quantum dots: The case of C132,” J. Phys. Chem. A 118(28), 5189–5195 (2014). 10.1021/jp502753a 24971474

[159] L. A. Falkovsky, “Optical properties of graphene and IV–VI semiconductors,” Phys. Usp. 51(9), 887 (2008). 10.1070/PU2008v051n09ABEH006625

[160] M. Orlita and M. Potemski, “Dirac electronic states in graphene systems: Optical spectroscopy studies,” Semicond. Sci. Technol. 25(6), 063001 (2010). 10.1088/0268-1242/25/6/063001

[161] S. Schöche *et al.*, “Optical properties of graphene oxide and reduced graphene oxide determined by spectroscopic ellipsometry,” Appl. Surf. Sci. 421, 778–782 (2017). 10.1016/j.apsusc.2017.01.035

[162] J. Weber, V. Calado, and M. Van De Sanden, “Optical constants of graphene measured by spectroscopic ellipsometry,” Appl. Phys. Lett. 97(9), 091904 (2010). 10.1063/1.3475393

[163] K. F. Mak *et al.*, “The evolution of electronic structure in few-layer graphene revealed by optical spectroscopy,” Proc. Natl. Acad. Sci. U. S. A. 107(34), 14999–15004 (2010). 10.1073/pnas.1004595107 20696939 PMC2930520

[164] R. M. Hochstrasser, “Two-dimensional spectroscopy at infrared and optical frequencies,” Proc. Natl. Acad. Sci. U. S. A. 104(36), 14190–14196 (2007). 10.1073/pnas.0704079104 17664429 PMC1964834

[165] A. Mohammed and A. Abdullah, “Scanning electron microscopy (SEM): A review,” in Proceedings of the 2018 International Conference on Hydraulics and Pneumatics (HERVEX), Băile Govora, Romania (2018).

[166] W. Zhou *et al.*, “Fundamentals of scanning electron microscopy (SEM),” in *Scanning Microscopy for Nanotechnology: Techniques and Applications* (Springer, 2007), pp. 1–40.

[167] H. Leamy, “Charge collection scanning electron microscopy,” J. Appl. Phys. 53(6), R51–R80 (1982). 10.1063/1.331667

[168] Z.-J. Wang *et al.*, “Direct observation of graphene growth and associated copper substrate dynamics by in situ scanning electron microscopy,” ACS Nano 9(2), 1506–1519 (2015). 10.1021/nn5059826 25584770

[169] W. Sohn, M. Kim, and H. W. Jang, “Atomic-scale insights into the 2D materials from aberration-corrected scanning transmission electron microscopy: Progress and future,” Small Sci. 4(2), 2300073 (2024). 10.1002/smsc.202300073 40212345 PMC11935267

[170] R. Zan *et al.*, “Metal−graphene interaction studied via atomic resolution scanning transmission electron microscopy,” Nano Lett. 11(3), 1087–1092 (2011). 10.1021/nl103980h 21271746

[171] K. Takahashi *et al.*, “In situ scanning electron microscopy of graphene growth on polycrystalline Ni substrate,” Surf. Sci. 606(7–8), 728–732 (2012). 10.1016/j.susc.2011.12.009

[172] J. D. Stoll and A. Kolmakov, “Electron transparent graphene windows for environmental scanning electron microscopy in liquids and dense gases,” Nanotechnology 23(50), 505704 (2012). 10.1088/0957-4484/23/50/505704 23165114

[173] O. Dyck *et al.*, “Placing single atoms in graphene with a scanning transmission electron microscope,” Appl. Phys. Lett. 111(11), 113104 (2017). 10.1063/1.4998599

[174] M. Wojcik *et al.*, “Graphene-enabled electron microscopy and correlated super-resolution microscopy of wet cells,” Nat. Commun. 6(1), 7384 (2015). 10.1038/ncomms8384 26066680 PMC4490578

[175] A. W. Robertson and J. H. Warner, “Atomic resolution imaging of graphene by transmission electron microscopy,” Nanoscale 5(10), 4079–4093 (2013). 10.1039/c3nr00934c 23595204

[176] A. Shalaby *et al.*, “Structural analysis of reduced graphene oxide by transmission electron microscopy,” Bulg. Chem. Commun. 47(1), 291–295 (2015).

[177] R. S. Pantelic *et al.*, “The application of graphene as a sample support in transmission electron microscopy,” Solid State Commun. 152(15), 1375–1382 (2012). 10.1016/j.ssc.2012.04.038

[178] M. Textor and N. de Jonge, “Strategies for preparing graphene liquid cells for transmission electron microscopy,” Nano Lett. 18(6), 3313–3321 (2018). 10.1021/acs.nanolett.8b01366 29799208

[179] A. Politano, G. Chiarello, and C. Spinella, “Plasmon spectroscopy of graphene and other two-dimensional materials with transmission electron microscopy,” Mater. Sci. Semicond. Process. 65, 88–99 (2017). 10.1016/j.mssp.2016.05.002

[180] R. Zan *et al.*, “Interaction of metals with suspended graphene observed by transmission electron microscopy,” J. Phys. Chem. Lett. 3(7), 953–958 (2012). 10.1021/jz201653g 26286426

[181] X. H. Liu *et al.*, “In situ transmission electron microscopy of electrochemical lithiation, delithiation and deformation of individual graphene nanoribbons,” Carbon 50(10), 3836–3844 (2012). 10.1016/j.carbon.2012.04.025

[182] Y. Lu *et al.*, “In situ electronic characterization of graphene nanoconstrictions fabricated in a transmission electron microscope,” Nano Lett. 11(12), 5184–5188 (2011). 10.1021/nl2023756 22026483 PMC3382988

[183] O. Lehtinen *et al.*, “Non-invasive transmission electron microscopy of vacancy defects in graphene produced by ion irradiation,” Nanoscale 6(12), 6569–6576 (2014). 10.1039/c4nr01918k 24802077

[184] C. Luo *et al.*, “In Situ transmission electron microscopy characterization and manipulation of two-dimensional layered materials beyond graphene,” Small 13(35), 1604259 (2017). 10.1002/smll.201604259 28783241

[185] T. Jiang and Y. Zhu, “Measuring graphene adhesion using atomic force microscopy with a microsphere tip,” Nanoscale 7(24), 10760–10766 (2015). 10.1039/C5NR02480C 26035717

[186] S. Eigler *et al.*, “Statistical Raman microscopy and atomic force microscopy on heterogeneous graphene obtained after reduction of graphene oxide,” J. Phys. Chem. C 118(14), 7698–7704 (2014). 10.1021/jp500580g

[187] N. Lindvall, A. Kalabukhov, and A. Yurgens, “Cleaning graphene using atomic force microscope,” J. Appl. Phys. 111(6), 064904 (2012). 10.1063/1.3695451

[188] R. Rasuli and M. Ahadian, “Mechanical properties of graphene cantilever from atomic force microscopy and density functional theory,” Nanotechnology 21(18), 185503 (2010). 10.1088/0957-4484/21/18/185503 20388969

[189] F. Lavini *et al.*, “Atomic force microscopy phase imaging of epitaxial graphene films,” J. Phys. Mater. 3(2), 024005 (2020). 10.1088/2515-7639/ab7a02

[190] L.-Y. Lin *et al.*, “Friction and wear characteristics of multi-layer graphene films investigated by atomic force microscopy,” Surf. Coat. Technol. 205(20), 4864–4869 (2011). 10.1016/j.surfcoat.2011.04.092

[191] T. Filleter and R. Bennewitz, “Structural and frictional properties of graphene films on SiC (0001) studied by atomic force microscopy,” Phys. Rev. B 81(15), 155412 (2010). 10.1103/PhysRevB.81.155412

[192] L. Weng *et al.*, “Atomic force microscope local oxidation nanolithography of graphene,” Appl. Phys. Lett. 93(9), 093107 (2008). 10.1063/1.2976429

[193] A. Giesbers *et al.*, “Nanolithography and manipulation of graphene using an atomic force microscope,” Solid State Commun. 147(9–10), 366–369 (2008). 10.1016/j.ssc.2008.06.027

[194] Y. Yao *et al.*, “Histogram method for reliable thickness measurements of graphene films using atomic force microscopy (AFM),” J. Mater. Sci. Technol. 33(8), 815–820 (2017). 10.1016/j.jmst.2016.07.020

[195] M. Pumera, “Graphene-based nanomaterials and their electrochemistry,” Chem. Soc. Rev. 39(11), 4146–4157 (2010). 10.1039/c002690p 20623061

[196] C. I. Justino, T. A. Rocha-Santos, and A. C. Duarte, “Review of analytical figures of merit of sensors and biosensors in clinical applications,” Trends Anal. Chem. 29(10), 1172–1183 (2010). 10.1016/j.trac.2010.07.008

[197] F. Cui and H. S. Zhou, “Diagnostic methods and potential portable biosensors for coronavirus disease 2019,” Biosens. Bioelectron. 165, 112349 (2020). 10.1016/j.bios.2020.112349 32510340 PMC7266610

